# TDP-43 nuclear retention is antagonized by hypo-phosphorylation of its C-terminus in the cytoplasm

**DOI:** 10.1038/s42003-025-07456-7

**Published:** 2025-01-28

**Authors:** Célia Rabhi, Nicolas Babault, Céline Martin, Bénédicte Desforges, Alexandre Maucuer, Vandana Joshi, Serhii Pankivskyi, Yitian Feng, Guillaume Bollot, Revital Rattenbach, David Pastré, Ahmed Bouhss

**Affiliations:** 1 Université Paris-Saclay, INSERM U1204, Univ Evry, Structure-Activité des Biomolécules Normales et Pathologiques (SABNP), Evry-Courcouronnes, France; 2 4P-Pharma, Campus Pasteur Lille, 59000 Lille, France; 3 SYNSIGHT, Evry, France

**Keywords:** Phosphorylation, Drug screening

## Abstract

Protein aggregation is a hallmark of many neurodegenerative disorders, including amyotrophic lateral sclerosis (ALS), in which TDP-43, a nuclear RNA-binding protein, forms cytoplasmic inclusions. Here, we have developed a robust and automated method to assess protein self-assembly in the cytoplasm using microtubules as nanoplatforms. Importantly, we have analyzed specifically the self-assembly of full-length TDP-43 and its mRNA binding that are regulated by the phosphorylation of its self-adhesive C-terminus, which is the recipient of many pathological mutations. We show that C-terminus phosphorylation prevents the recruitment of TDP-43 in mRNA-rich stress granules only under acute stress conditions because of a low affinity for mRNA but not under mild stress conditions. In addition, the self-assembly of the C-terminus is negatively regulated by phosphorylation in the cytoplasm which in turn promotes TDP-43 nuclear import. We anticipate that reducing TDP-43 C-terminus self-assembly in the cytoplasm may be an interesting strategy to reverse TDP-43 nuclear depletion in neurodegenerative diseases.

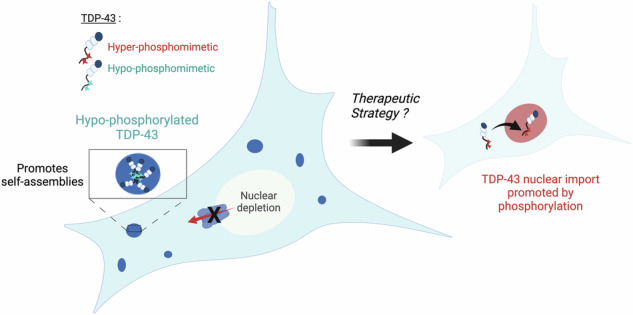

## Abstract (web summary)

Shedding light onto the role of phosphorylation in TDP-43 cytoplasmic mis-localization.

## Introduction

**Fig. 1:**
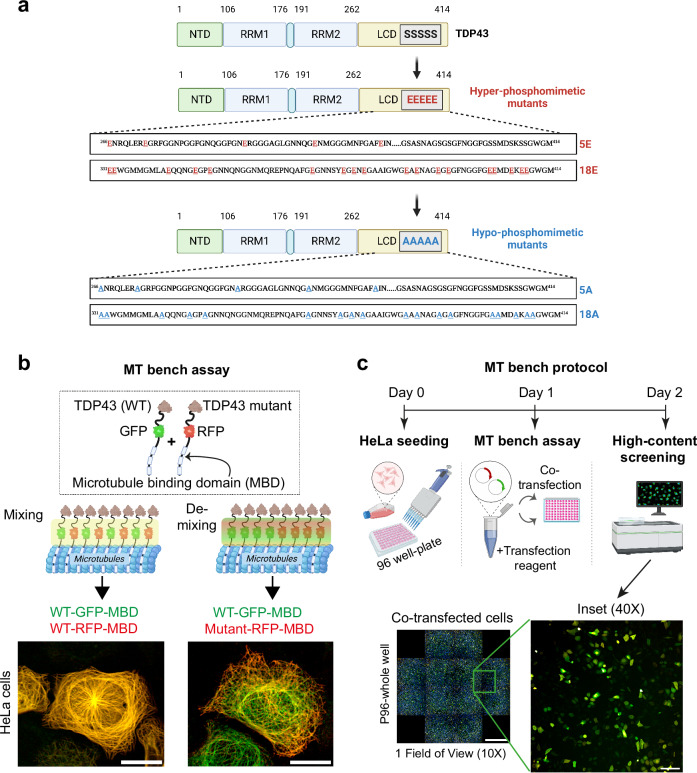
Schematic representation of the experimental approach for identifying kinase and phosphatase inhibitors targeting the TDP-43 C-terminus self-assembly in the cytoplasm. **a** Schematic representation of TDP-43 hyper- or hypo-phosphomimetic mutants. **b** Description of the MT bench technology which is designed to probe the mixing between wild-type and mutant TDP-43 along microtubules in HeLa cells co-expressing the indicated protein fusions. Scale bar: 20 μm. **c** Automatic detection of single-cell fluorescence via an automatic HCS imager in 96-well plates for measuring the mixing/de-mixing between two proteins along the microtubule network. Scale bar: 100 μm. Zoom scale bar: 50 μm. This panel was created in BioRender. Rattenbach, R. (2025) https://BioRender.com/r85f184.

TDP-43 is an RNA-binding protein expressed in all human tissues that mainly participates in nuclear functions associated with mRNA processing^1,2^. TDP-43 exhibits a long self-adhesive domain of low complexity (LCD) in its C-terminus that allows the formation of reversible cellular compartments with liquid-like properties^3–5^ (Fig. 1a). Additional contributions also come from its structured self-associating N-terminal domain^6^ and the cooperative association of its two RNA-recognition Motifs (RRM-1 and RRM-2) to GU-rich repeats^3,7^. TDP-43 is involved in certain neurodegenerative diseases, such as amyotrophic lateral sclerosis (ALS) and frontotemporal lobar degeneration (FTLD)^8,9^. Indeed, both the identification of pathological mutations, notably in the unstructured C-terminal domain of TDP-43 and the occurrence with which TDP-43 forms cytoplasmic inclusions in the affected neurons explain the particular interest of the scientific community in this RNA-binding protein^7,10^. Accordingly, TDP-43 is considered a major target with the aim of preventing its cytoplasmic aggregation^11^ or restoring its nuclear location^12–14^. These two aims are intertwined since TDP-43-rich cytoplasmic inclusions deplete the pool of nuclear TDP-43, which in turn prevents TDP-43 self-regulation of its own expression^15,16^. A decreased concentration of nuclear TDP-43 may thus induce a toxic feedforward mechanism to generate additional TDP-43-rich cytoplasmic inclusions.

It has been shown that the expression of TDP-43 pathological mutants can be associated with defective stress granule assembly and nuclear depletion TDP-43^17,18^. TDP-43 aggregation is thought to take place after an aberrant transition from a liquid- to solid-like state in TDP-43 condensates^19^, which is promoted by pathological mutations in the self-adhesive C-terminal domain of TDP-43^20^. The formation of solid-like TDP-43 aggregates can be prevented by the presence of RNA in the nucleus, which acts as a chaperone^19,21^, notably, in the case of TDP-43, by binding to GU-rich sequences^22^. In addition, protein chaperones such as heat shock proteins can also contribute to preserving the reversibility of TDP-43 condensate^23–25^. Besides RNA and protein chaperones, TDP-43 solubility may be preserved by post-translational modifications, notably phosphorylation^26,27^. In this study, we focus our attention on multiple phosphorylation sites located in the C-terminal domain of TDP-43, which regulate the capacity of TDP-43 to form liquid-like compartments^28^.

The role of TDP-43 phosphorylation in its cytoplasmic location and aggregation remains a matter of debate. TDP-43 phosphorylation has been associated with the presence of TDP-43 in the cytoplasm and the formation of inclusions^29–32^, with the detection of phosphorylated TDP-43 in cytoplasmic aggregates identified in affected neurons^33,34^. Consistently, the activity of protein kinases such as CK1^35,36^, TTBK1^37^, or GSK3b^38^, as well as phosphomimetic mutants, were reported to increase the presence of TDP-43 in the cytoplasm. Phosphorylation can thus be considered as a driver of TDP-43 nuclear mislocalization and of its cytoplasmic aggregation. However, in affected neurons, these phosphorylation events may occur downstream of TDP-43 aggregation and its truncation^39^. In addition, in other studies, multiple TDP-43 phosphorylation sites in the C-terminal domain prevented TDP-43 to form aggregates^30^. The formation of liquid droplets in vitro was also affected by phosphomimetic mutants compared to wild-type TDP-43^28^. Therefore, TDP-43 C-terminus phosphorylation may also antagonize TDP-43 aggregation by decreasing its propensity to form liquid droplets. In keeping with the latter hypothesis, the recruitment of TDP-43 in stress granules can be inhibited by the hyperphosphorylation of TDP-43 C-terminus^28^, and/or TDP-43 phosphorylation can be prevented by the recruitment of TDP-43 in stress granules^40^. Stress granules are cytoplasmic mRNA-rich liquid-like condensates that are formed in response to a large variety of cellular stress. The recruitment of TDP-43 into mRNA-rich stress granules may provide a means to keep cytoplasmic TDP-43 soluble, notably by preserving TDP-43 interaction with mRNA^19,40^. However, this model is still debated. On the one hand, stress granules can be considered as crucibles to promote TDP-43 aggregation^41–43^. On the other hand, stress granules may have no influence^17,18^ or antagonize the formation of TDP-43 aggregates^40,44^. In summary, no real consensus has been reached on the consequences of TDP-43 phosphorylation on its subcellular distribution and the formation of TDP-43 cytoplasmic inclusions.

Here we develop a new approach based on an entirely automated analysis of single cells at high resolution using a confocal HCS imager. The HCS imager also operates in confocal mode at high resolution with water-immersed lenses. High resolution enabled us to score TDP-43 self-assemblies controlled by the phosphorylation of the C-terminal domain in the cytoplasm using microtubules as nanoplatforms (MT bench assay^7,45^). The “MT bench” approach allows the screening of the self-association of TDP-43 in the presence of mRNA. This method is sufficiently sensitive to detect the effect of TDP-43 point mutations on its higher-order assembly in a cellular context^7^, provided that the experimental conditions tested do not affect the microtubule network. After validation with phosphomimetic controls, we demonstrated the ability of our method to quantify TDP-43-specific assemblies by detecting the aggregation of TDP-43 under arsenite stress^46,47^. While TDP-43 hyper-phosphomimetic mutants are not recruited in stress granules during acute stress^28,43^, we did measure significant recruitment of TDP-43 hyper-phosphomimetic mutants after mild stress. Our results indicate that intermolecular interactions involving RRM domains, most likely through disulfide cysteine bridges or acetylation of Lysine residues^46,47^, prevent the recruitment of TDP-43 hyper-phosphomimetic mutants in stress granules but only under acute stress conditions. Having demonstrated the potential of our approach, we screened and selected 14 out of 98 kinase inhibitors and 2 out of 36 phosphatase inhibitors that significantly modulate the phosphorylation-dependent TDP-43 self-assembly when comparing wild-type TDP-43 mixing with TDP-43 hyper- or hypo-phosphomimetic protein mutants. We then revealed that TDP-43 hypo-phosphomimetic mutants and selected kinase inhibitors negatively regulate TDP-43 nuclear retention, which may be considered as a negative contribution in TDP-43 pathologies^14,48–50^. Interestingly, kinase and phosphatase inhibitors, respectively, increase and decrease the cytoplasmic condensation of a TDP-43 mutant (G146A), which is defective in its cooperative association with GU-rich mRNA^7^. Altogether, our stringent large-scale screen and functional cellular assays enabled the identification of three kinases and two phosphatase inhibitors that target phosphorylation-dependent TDP-43 self-assembly. We also established that the phosphorylation-dependent self-assembly of the C-terminal domain is critical to keeping TDP-43 in the nucleus of HeLa cells and of a mouse motor neuron cell line (NCS-34). Together, our data provide the basis for further investigations into how TDP-43 phosphorylation may interfere with pathological mutations identified in the C-terminus of TDP-43 and whether interfering with TDP-43 self-assembly in the cytoplasm may correct the negative impact of TDP-43 pathological mutations.

## Results

### An automated pipeline to detect phosphorylation-dependent TDP-43 self-assembly in the cytoplasm

**Fig. 2:**
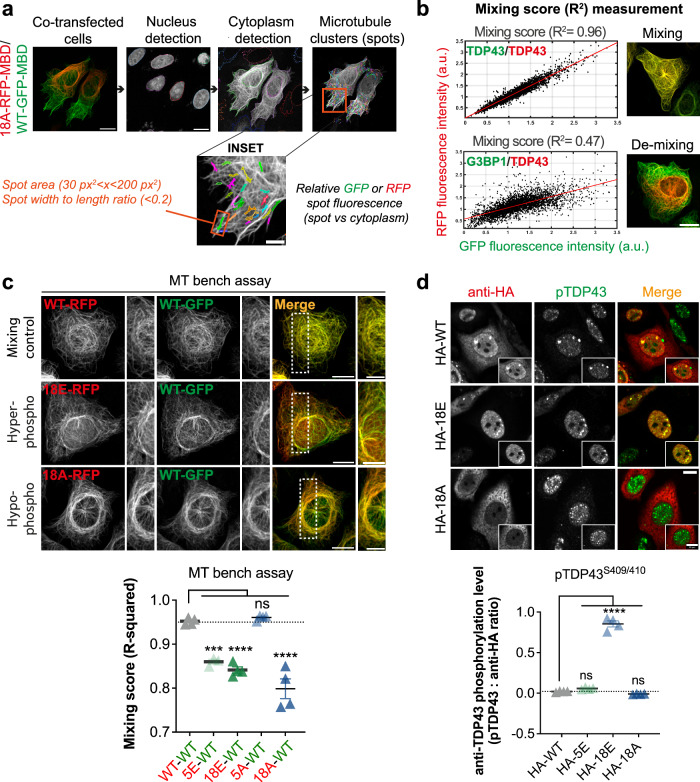
Hypo- and hyper phosphomimetic mutants significantly demix with wild-type TDP-43. **a** Presentation of the automatic detection scheme allowing to measure fluorescence intensities in spots along the microtubules network in HeLa cells co-expressing wild-type and mutant TDP-43 fused to a microtubule binding-domain (MBD) by using the Harmony software. In each “microtubule” spot, the intensity of RFP and GFP was measured as well as in the surrounding cytoplasm. Scale bar: 50 μm. Zoom scale bar: 20 μm. **b** Left panel: Scatter plot of the relative GFP and RFP fluorescence intensity (spots versus cytoplasm) detected in all HeLa cells present in a single well under indicated conditions. The *R*-squared (*R*^2^) value indicates to which extent the scatter plot distribution can be fitted using linear regression (*R*² = 1: perfect mixing between the two proteins). Right panel: images of Hela cells showing that TDP-43 is well mixed with itself (*R*² = 0.96) but not with G3BP-1 (*R*² = 0.47). Scale bar: 50 μm. **c** Representative images of HeLa cell co-expressing indicated proteins used to measure their mixing along the microtubule network. Scale bar: 50 μm. Zoom scale bar: 30 μm. Lower panel: Mixing score (R² values) measured in HeLa cells expressing wild-type TDP-43 and the indicated phosphomimetic mutants (see Fig. 1a). Error bars indicate SEM and the asterisks indicate statistical significance with ***p < 0.001, ****p < 0.0001, ns. non-significant, as measured by ANOVA test from *n* = 4 wells (each dot represents one well). **d** Images of HeLa cells expressing indicated HA-tagged protein and immune-stained with an anti-phospho TDP-43 antibody (Ser409/410). Note the co-localization of the hyper-phosphomimetic TDP-43 mutant and the anti-phospho TDP-43 in the nucleoplasm and some nuclear granules. Scale bars: 20 μm. Lower panel: Relative increase in cellular anti-phospho TDP-43 (Ser409/410) fluorescence intensity with respect to anti-HA intensity in HeLa cells expressing indicated HA-tagged TDP-43 phosphomimetic mutant. Note the non-recognition of the 5E mutant by the anti-phospho TDP-43 since Ser409/410 were not mutated into Glu residues in this mutant. Error bars indicate SEM and the asterisks indicate statistical significance with ****p < 0.0001, ns non-significant, as measured by ANOVA test from *n* = 4 wells (each dot represents one well).

Various screens have been implemented to detect how small molecules can interfere with liquid–liquid phase separation (LLPS) of RNA-binding proteins or stress granule formation^51,52^. In addition, TDP-43-centered studies have also focused on identifying small compounds to alter the recruitment of TDP-43 in stress granules^41^ or RNA recognition^53^. However, even if the role of TDP-43 phosphorylation is recognized as essential for its physiological mRNA-related functions or its pathological aggregation^39^, though with opposite views, large-scale studies aiming at identifying kinase and phosphatase inhibitors are scarce^54^. Here, we opted for a screen based on the self-assembly of TDP-43 in the cytoplasm, whose loss of reversibility contributes to the formation of TDP-43-rich inclusions in the cytoplasm. It also promotes the loss of TDP-43 nuclear functions. To identify specific kinase or phosphatase inhibitors, our approach detects a variation of miscibility between wild-type TDP-43 and a hyper- or hypo-phosphomimetic mutant in the cytoplasm. To this end, we use a new technology, the MT bench^45^, to measure the mixing/de-mixing of two proteins brought onto the microtubule network in cells by fusing them to a microtubule-binding domain (MBD). In addition, the two proteins are either RFP- or GFP-labeled to distinguish them from each other (Fig. 1b, Table S1). The approach also utilizes fully automated robots for cell manipulation and drug molecule handling, as well as automatic acquisition/analysis of high-resolution images in confocal mode in 96-well plates (Figs. 1c and 2a-c and see the “Methods” section). We have already validated this approach in the case of TDP-43 by detecting the formation of mRNA-rich reversible compartments along the microtubule network^45^. The sensitivity of our method enables us to detect whether single mutations interfere with the mixing of wild-type TDP-43^7^. In addition, using our pipeline, we further controlled that point mutations that impair either the self-association N-terminal domain or its cooperative association to mRNA lead to the demixing of mutated TDP-43 with wild-type TDP-43. As expected, the two TDP-43 mutants strongly demixed with wild-type TDP-43 (Supplementary Fig. 1)^7^. To undertake our analysis, we measured the mixing/de-mixing of wild-type TDP-43 with hypo- or hyper-phosphomimetic mutants, which contain several serine substitutions for alanine or glutamic acid residues in the C-terminal domain of TDP-43 similar to those used in a previous study^28^ (Fig. 1a). However, though our assay is specific to C-terminus-dependent TDP-43 self-assembly, we cannot distinguish whether kinase inhibitors directly change the phosphorylation status of TDP-43 C-terminus or regulate the mixing with TDP-43 phosphomimetic by an indirect mechanism (for instance by acting on chaperone protein activity, RNA helicase activity, mRNA translation, etc.).

### Both hyper- and hypo-phosphomimetic mutants significantly de-mix with wild-type TDP-43, but in different ways

To explore whether TDP-43 phosphorylation interferes with self-assembly in the cytoplasm, we generated several full-length hyper- and hypo-phosphomimetic TDP-43 mutants (Fig. 1a, Tables S2–S4). We chose to mutate the N-terminus LCD domain of TDP-43 by using two complement constructions, the first construction with 5 mutations located downstream of RRM domains (from Serine-266 to Serine-317) and the second construction with 18 mutations (from Serine-331 to Serine-410). 18E/18A includes 12 mutated serine residues used in previous reports 12E/12A^28^. Phosphomimetic mutants are useful for mimicking TDP-43 phosphorylation in the C-terminus, although multiple mutations can change the structure and behavior of the C-terminus compared to native phosphorylation. Nevertheless, these mutations modify the biophysical properties of the C-terminus and prevent its modification by kinases or phosphatases. Thus, phosphomimetic mutants can be used to screen kinase and phosphatase inhibitors that regulate the higher-order assembly of TDP-43 through its unstructured self-adhesive C-terminus. To determine the extent to which phosphomimetic mutants can mimic native TDP-43 phosphorylation, HeLa cells expressing phosphomimetic mutants were stained with an anti-phospho TDP-43 antibody (Ser 409/410). In contrast with the hypo-phosphomimetic mutants, the anti-phospho TDP-43 antibody clearly recognizes hyper-phosphomimetic mutants (18E, 12E) when residues 409/410 were included among the mutated residues, but not when absent (5E) (Fig. 2d and Supplementary Fig. 2a). This result suggests that the phospho-epitope is strongly mimicked by the hyper-phosphomimetic mutant, in agreement with a previous report^55^. We also investigated whether the recognition of the phosphomimetics is still observed if serine residues were mutated into aspartate residues (D) instead of glutamic acid residues (E) (Supplementary Fig. 2a). Thus, both acidic residues can be used to mimic the phosphomimetic feature since no effect of the nature of acidic side chain was observed.

Using the MT bench assay, we then explored whether phosphorylation in the C-terminus regulates TDP-43 self-assembly, as found in vitro^28^. After confirming the mixing of wild-type TDP-43 with itself or its de-mixing with two RNA-binding proteins that do not interact with wild-type TDP-43 (HuR and G3BP1) (Fig. 2b and Supplementary Fig. 8a), we measured the mixing of wild-type TDP-43 with several hypo- or hyper-phosphomimetics targeting the C-terminal domain in HeLa cells (Fig. 2b, c). We observed a de-mixing of wild-type TDP-43 with both the hypo- and hyper-phosphomimetic mutants (Fig. 2c). Importantly, the hyper- and hypo-phosphomimetic mutants generated two different compartments on microtubules that, accordingly, strongly de-mixes with each other but not with themselves (Supplementary Fig. 3b). The hyper-phosphomimetic mutant (18E) generated less dense compartments than those of the hypo-phosphomimetic mutant (18A) (Supplementary Fig. 3a), supporting the decrease in TDP-43 self-assemblies upon phosphorylation of the C-terminal domain^28^. Additionally, we measured the de-mixing of different phosphomimetic mutants with wild-type TDP-43 (Fig. 2c). As expected, the hyper- and hypo-phosphomimetic mutant containing the most mutations, 18E and 18A, respectively, induced the strongest de-mixing with wild-type TDP-43. Interestingly, hypo-phosphomimetic mutations downstream of the RRM2 (5A) did not induce any significant de-mixing with wild-type TDP-43, unlike the 5E mutant bearing the respective hyper-phosphomimetic mutations. It is possible that the serine residues mutated in 5A/5E proteins are dephosphorylated in wild-type TDP-43 under our experimental conditions.

### Hyper-phosphomimetic mutations hinder TDP-43 translocation

Having validated the use of phosphomimetic mutants for the MT bench assay, we considered functional assays as complementary screens to increase the stringency of compound selection. Using HA-tagged phosphomimetic mutants, we sought putative phenotypes that could be associated with TDP-43 phosphorylation, including TDP-43 translocation, recruitment in stress granules, condensation in the cytoplasm, and nuclear mRNA splicing in HeLa cells (Supplementary Fig. 4).

**Fig. 3:**
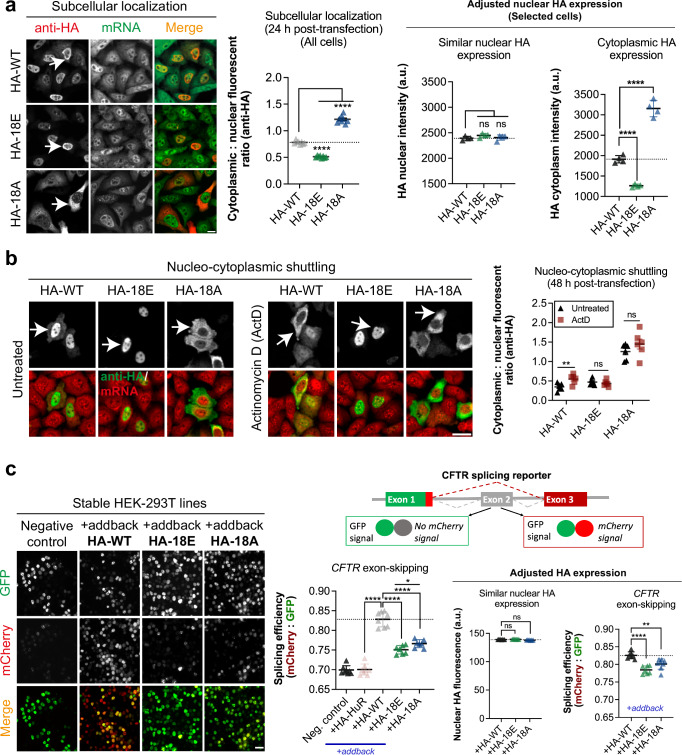
Mimicking the hypo-phosphorylation of TDP-43 C-terminus antagonizes its nuclear retention in HeLa cells. **a** Left panel: Subcellular distribution of indicated HA-tagged phosphomimetic mutants expressed in HeLa cells 24 h after transfection. Poly(A) mRNA in green; anti-HA in red. Middle panel: Automatic measurement of the ratio of the HA-tagged TDP-43 cytoplasmic to nuclear level under indicated conditions. Right panel: Same measurement as the middle panel with a selection of cells displaying similar nuclear TDP-43 levels. Each dot represents the mean value of a single well (96-well plate). Error bars indicate SEM and the asterisks indicate statistical significance with ****p < 0.0001, ns. non-significant, as measured by ANOVA test from *n*  =  6 wells. Arrows indicate representative cells. Scale bar: 20 μm. **b** Same as (**a**) 48 h post-transfection in HeLa cells treated with Actinomycin D (ActD) or not to promote TDP-43 translocation into the cytoplasm. Error bars indicate SEM and the asterisks indicate statistical significance with **p < 0.01, ns. non-significant, as measured by ANOVA test from *n* = 8 wells. Arrows indicate representative cells. Scale bar: 20 μm. **c** Analysis of the skipping of *CFTR* exon 9, which is controlled by TDP-43, using the indicated minigene reporter in stable shRNA HEK cells that no longer expressed endogenous TDP-43 and after adding back the expression of HA-tagged TDP-43 mutants. Left panel: Representative image of stable shRNA HEK cells under indicated conditions. Upper right panel: Splicing reporter used to detect the skipping of *CFTR* exon 9. Lower right panel: Quantitative analysis of the splicing efficiency and anti-HA fluorescence intensity under the indicated condition at the single cell level. Each dot represents the average values in selected HEK cells in a single well. Quantitative analysis was performed without any selection (left) or with cells expressing the same level of HA-tagged protein in the nucleus as shown in the panel (right). Error bars indicate SEM and the asterisks indicate statistical significance with *p < 0.05 as measured by unpaired Student’s *t*-test and ****p < 0.0001, ns non-significant, as measured by ANOVA test from *n* = 8 wells. Scale bar: 50 μm.

We noticed that 24 h after transfection, hypo-phosphomimetic mutants strikingly accumulated in the cytoplasm, while hyper-phosphomimetic mutants remained in the nucleus even more than wild-type TDP-43 (Fig. 3a). The results were surprising since similar mutations did not affect the subcellular location of TDP-43 in a previous report^28^. Puzzled about this discrepancy, we further tested the dynamics of the nucleocytoplasmic shuttling of wild-type and mutant TDP-43. To this end, we chose to use actinomycin D (ActD) to stop transcription and subsequently promote the translocation of TDP-43 in the cytoplasm^48^, which may be less intrusive than using hormone-inducible nuclear export assay requiring TDP-43 fusion with a hormone-binding domain^28^. We also increased the time after transfection prior to ActD treatment (48 h instead of 24 h), which led to an increased presence of wild-type TDP-43 in the cytoplasm^48^. In contrast, the subcellular distribution of the hyper- and hypo-phosphomimetics mutants (18E, 18A) remained unchanged (Fig. 3b).

As some of the phosphomimetic mutants tested also included mutations in a short conserved region (a.a. 316–346) that possibly adopts an α-helix secondary structure to form weak homo-oligomeric contacts that could promote TDP-43 condensation^20^, we tested TDP-43 mutants with no mutation in the putative short helix. Similar results were obtained regarding the subcellular location of TDP-43 phosphomimetic mutants, whether the short helix was mutated or not (Supplementary Fig. 2b). Finally, we also probed whether mutations into aspartate residues instead of glutamic acid residues would affect the results, but it was not the case (Supplementary Fig. 2b).

We then explored whether phosphomimetic mutations in the C-terminus domain also interfere with TDP-43 nuclear functions. To this end, we used a well-characterized splicing reporter in which the presence of an intronic 12 GU repeats recognized by TDP-43 controls the excision of exon 9 in the *CFTR* gene^56,57^ (Fig. 3c, Supplementary Fig. 5a and b). We reported only splicing events occurring in similar windows of mRNA reporter expression (GFP signal) for all the mutants and controls (Supplementary Fig. 5c). Using shRNA-treated HEK cells that no longer express TDP-43, we found that expression of shRNA-resistant wild-type TDP-43-HA restored the skipping of exon 9 (Tables S5 and S6), as expected. However, the expression of either HA-tagged hypo- or hyper-phosphomimetic mutants failed to completely restore exon 9 excision, albeit to a greater extent than HuR, an RNA-binding protein used as a negative control (Fig. 3c). However, a significant difference in the response of the splicing efficiency was observed in the presence of HA-tagged hypo- or hyper-phosphomimetic mutants (Fig. 3c). Our results were consistent with a putative role of the TDP-43 C-terminal domain in *CFTR* gene splicing^58^. To ensure that differences in the subcellular location of TDP-43 mutants at the single-cell level did not bias the results, we also selected cells with very similar nuclear HA-tagged TDP-43 expression at the single-cell level (Fig. 3c). Once again, the results suggest that, under our experimental conditions, phosphorylation/dephosphorylation events occurring in the TDP-43 C-terminus domain may contribute to the regulation of *CTFR* mRNA splicing in the nucleus by differentially regulating TDP-43 self-assembly or its interaction with mRNA splicing partners^58^. However, further investigations are required to further generalize these results in endogenous splicing events.

As there is emerging evidence that ALS-associated TDP-43 mutations further enhance its accumulation in mitochondria, and that inhibiting its translocation through the inner mitochondrial membrane can prevent neurotoxicity^59,60^, we also probed the differential presence of TDP-43 phosphomimetic mutants in mitochondria. Using an anti-TOM40 antibody to detect mitochondria in HeLa cells, we measured the enrichment of TDP-43 in mitochondria versus cytoplasm. The results indicate a slight but significant increase in the enrichment of the hyper-phosphorylation mutant, 18E, in mitochondria compared to wild-type TDP-43 and the hypo-phosphorylation mutant, 18A (Supplementary Fig. 6a–c). We then analyzed whether the expressions of TDP-43-variants in our experimental conditions differentially increase cell toxicity. When compared to wild-type TDP-43, none of these mutations did significantly affect the metabolic integrity or cell viability, as shown by both MTT and lactate dehydrogenase (LDH) release viability assays (Supplementary Fig. 6d–f). However, we noticed a non-significant increase in toxicity in cells expressing the hyper-phosphorylation mutant, 18E, compared to TDP-43 WT and 18A. An increased toxicity in cells expressing 18E, if any, may be related to its recruitment in mitochondria (Supplementary Fig. 6c) but also possibly to its nuclear retention (Fig. 3a).

### TDP-43 hyper-phosphomimetic mutant promotes RRM aggregation during acute oxidative stress, which prevents its recruitment in stress granules but only after acute stress

**Fig. 4:**
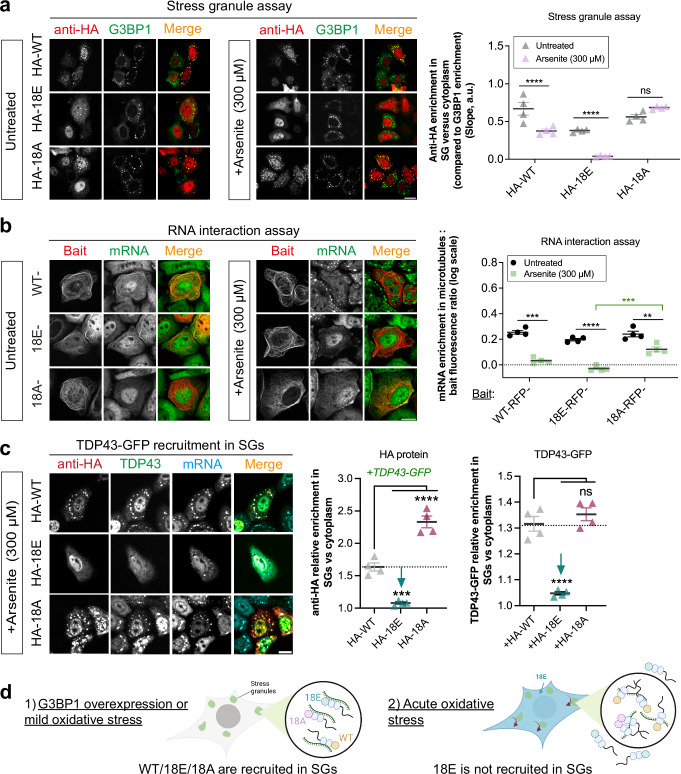
Hyper-phosphomimetic TDP-43 mutant is not recruited in stress granules but only under acute oxidative stress. **a** Left panel: Representative images of HeLa cells expressing HA-tagged phosphomimetic mutants and G3BP1-GFP with or without arsenite (300 µM, 1 h). Right panel: Automatic quantification of the recruitment of HA-tagged TDP-43 mutants in stress granules. Each dot represents the mean slope of the SG/Cytoplasm enrichment of HA versus GFP fluorescence measured over all stress granules detected in a single well (96-well plate). When the slope value is 0, there is no HA enrichment in SGs. Error bars indicate SEM and the asterisks indicate statistical significance with *p < 0.05, **p < 0.01, ****p < 0.0001, ns non-significant, as measured by Student’s *t*-test from *n*  =  4 wells. Scale bar: 50 μm. **b** Left panel: Representative images of HeLa cells expressing the indicated TDP-43 mutants fused to microtubule-binding domain to analyze the recruitment of mRNA along microtubules in the presence or absence of arsenite (300 µM, 1 h). Right panel: Relative recruitment of mRNA along microtubules for indicated proteins used as baits. Each dot represents the mean value over all stress granules detected in a single well (96-well plate). Note the absence of mRNA along microtubules with the hyper-phosphomimetic mutant (18E) after arsenite treatment. Error bars indicate SEM and the asterisks indicate statistical significance with **p < 0.01, ***p < 0.001, ****p < 0.0001, ns non-significant, as measured by Student’s *t*-test from *n*  =  4 wells. Scale bar: 50 μm. mRNA was detected by in situ hybridization with a fluorescent poly-dT probe. **c** Left panel: Representative images of HeLa cells expressing indicated HA-tagged TDP-43 mutants and wild-type TDP-43 in the presence of arsenite. Scale bar: 50 μm. Center panel: Measurement of the recruitment of HA-tagged TDP-43 mutant and wild-type TDP-43 in stress granules. Right panel: Measurement of the SG/Cytoplasm enrichment of anti-HA or GFP fluorescence in HeLa cells were co-transfected with GFP-tagged wild-type TDP-43 and HA-tagged wild-type or phosphomimetic mutants, as indicated. Wild-type TDP-43-GFP is no longer recruited in stress granules in arsenite-treated HeLa cells expressing the hyper-phosphomimetic mutant (18E). Error bars indicate SEM and the asterisks indicate statistical significance with ***p < 0.001, ****p < 0.0001, ns non-significant, as measured by ANOVA test from *n*  =  4 wells. **d** Schematic view of the differential recruitment of TDP-43 in stress granules regulated by the phosphorylation status of TDP-43 C-terminus domain and the level of oxidative stress.

To investigate whether TDP-43 phosphorylation controls its recruitment in mRNA-rich stress granules, HeLa cells expressing wild-type or mutated TDP-43 were subjected to arsenite (NaAsO_2_) stress^61^. Stress granules were detected using an automated pipeline using the HCS imager (Supplementary Fig. 7a) and G3BP1-GFP as a marker^62^. In our hands, 300 µM was the lowest arsenite concentration that can induce a robust formation of stress granules in the cytoplasm of a large majority of HeLa cells. Our results showed that TDP-43 hyper-phosphomimetic mutants were not recruited to stress granules, in agreement with a previous report^28^. The recruitment of wild-type TDP-43 in stress granules was also impaired, but to a lesser extent than the hyper-phosphomimetic mutant (18E), while the recruitment of the hypo-phosphomimetic mutant remained significant (Fig. 4a, Supplementary Fig. 7b, c). However, under mild oxidative stress conditions such as exposing cells to 300 µM hydrogen peroxide (H_2_O_2_) combined with puromycin^63^ or simply overexpressing G3BP1 to trigger stress granule assembly^62^, an efficient recruitment of TDP-43 hyper-phosphomimetics in stress granules was observed. This result questions whether the level of stress negatively regulates the recruitment of TDP-43 in stress granules. To address this point, we used an elevated concentration of H_2_O_2_ (1 mM) which nearly totally prevented the recruitment of TDP-43 hyper-phosphomimetic mutants in stress granules, but not that of TDP-43 hypo-phosphomimetic mutant (Supplementary Fig. 7d). Of note, the results obtained were similar regardless of whether D or E residues were used to mimic phosphorylation and whether serine residues in the C-terminus sequence (a.a. 316–346) that could form an α-helix^56^ were mutated (Supplementary Fig. 2c, mutants 12E and 12D). These data suggest that the non-recruitment of TDP-43 hyper-phosphomimetic mutants in stress granules relies on acute oxidative stress conditions rather than an inherent impossibility of phosphorylated TDP-43 to be recruited in stress granules.

Since stress granules contain non-polysomal mRNAs, we considered whether the differential recruitment of TDP-43 mutants in stress granules relies on a differential affinity of TDP-43 mutants for mRNA, which could depend on stress level. To this end, we used an adapted version of the MT bench method, which enables us to detect the binding of endogenous mRNAs to RBPs^64^. Briefly, a bait is brought onto microtubules, similar to our approach for measuring the mixing between two different proteins (Fig. 1b). Next, we measured the amount of endogenous mRNA recruited onto microtubules by wild-type or mutant TDP-43 used as baits. The quantitative measurements indicate that both TDP-43 hyper- and hypo-phosphomimetic mutant (18A and 18E) bind to mRNA significantly (mRNA enrichment > 1; Fig. 4b). This result is in line with a previous report using the EMSA approach in vitro which showed that recombinant hyper- and hypo-phosphomimetic mutants bind to RNA^28^. However, we noticed that the binding of 18E to mRNA in cells is slightly lower than that of wild-type TDP-43 and TDP-43 hypo-phosphomimetic mutant (18A) (Fig. 4b). However, under arsenite stress, we observed a sharp decrease in the recruitment of mRNA with the hyper-phosphomimetic mutant in contrast to the hypo-phosphomimetic mutant, which may explain the non-recruitment of the hyper-phosphomimetic mutant in stress granules upon arsenite exposure. One possible explanation is that disulfide cysteine bridges or acetylation of lysine residues in the RRM domains could impair RNA-binding and promote the accumulation of insoluble TDP-43 in cells exposed to acute oxidative stress, as reported in the literature^46,47^.

Therefore, we utilized the MT bench assay to probe the TDP-43 aggregation process after arsenite stress (Supplementary Fig. 8a, b). The de-mixing of TDP-43 with G3BP1 and HuR strongly increased in the presence of arsenite, which may be due to TDP-43 aggregation separately from G3BP1 or HuR. Interestingly, both the hypo- or hyper-phosphomimetic mutants were better mixed with wild-type TDP-43 in the presence of arsenite, compared to HuR and G3BP1, which should occur in the case of intermolecular TDP-43 RRM aggregation. Furthermore, we designed an experiment to investigate whether TDP-43 RRMs were specifically involved in this aggregation process. When TDP-43 RRM1/2 fragments were used as baits on microtubules, they failed to recruit TDP-43 hyper- and hypo-phosphomimetic mutants under normal conditions (Supplementary Fig. 9a, b). However, under arsenite stress, the hyper-phosphomimetic mutant was recruited onto microtubules, but not the hypo-phosphomimetic mutant.

To further explore this hypothesis without using the MT bench assay, we examined whether GFP-tagged wild-type TDP-43 could be routed out of stress granules upon aggregation with a hyper-phosphomimetic mutant after arsenite stress (Fig. 4c). In contrast with the hypo-phosphomimetic mutant (18A), GFP-tagged wild-type TDP-43 is indeed not recruited to stress granules in cells co-expressing GFP-tagged wild-type TDP-43 and the HA-tagged hyper-phosphomimetic mutant (18E) (Fig. 4c).

Taken together, these results demonstrate the capability of the MT bench assay to evaluate TDP-43 self-assemblies and suggest a higher propensity of TDP-43 hyper- than hypo-phosphomimetic mutants for RRM-mediated aggregation under acute oxidative stress (Fig. 4d, Supplementary fig. 9a, b). However, both hypo- and hyper-phosphomimetic mutants bind to mRNA and are recruited in stress granules without environmental oxidative stress (G3BP1 expression) or under mild oxidative stress conditions (H_2_O_2_ ≤ 300 µM).

### Identification of kinase and phosphatase inhibitors that can interfere specifically with TDP-43 self-assembly mediated by the C-terminal domain in the cytoplasm

**Fig. 5:**
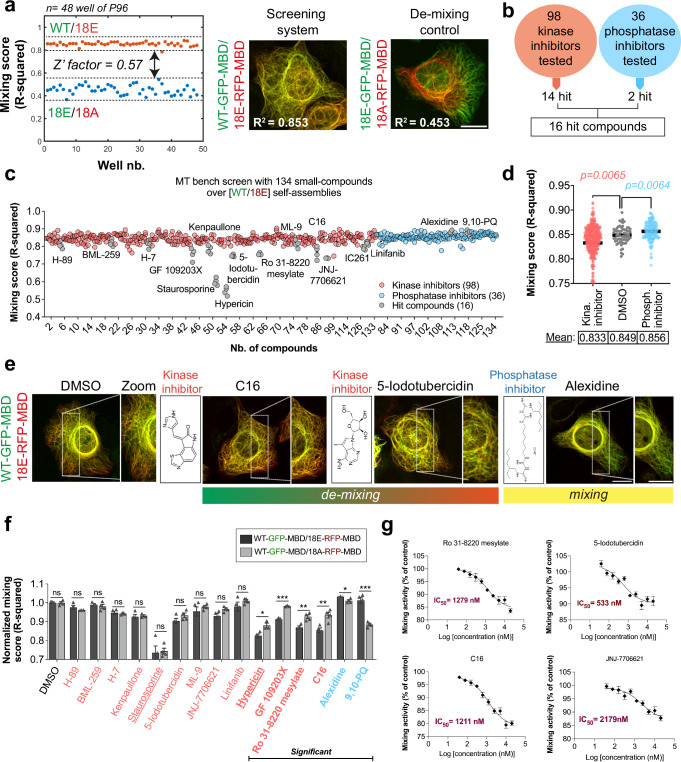
Identification of kinase/phosphatase inhibitors affecting TDP-43 C-terminus self-assembly in the cytoplasm. **a** Left panel: Mixing score between wild-type TDP-43 and 18E mutant and between 18E and 18A mutants in a 96-well plate. The measured *Z*’ factor is 0.57, which corresponds to a very good assay for compounds decreasing the mixing score. Right panel: Representative image of the mixing of indicated proteins along the microtubule networks in HeLa cells. Scale bar: 50 μm. **b** Summary of the results of the primary screen. **c** Scatter plot representing the mixing score for all the tested compounds. Red, kinase inhibitors, Blue: Phosphatase inhibitors, Gray: hits. HeLa cells were treated for 4 h with 10 µM of the indicated compounds. **d** Scatter plot of the mixing score for treatments with DMSO (control), kinase, and phosphatase inhibitors. Globally, kinase inhibitors decrease while phosphatase inhibitors increase the mixing of the hyper-phosphomimetic mutants (18E) with wild-type TDP-43. *p* values are indicated. ANOVA test. **e** Representative image of the mixing of wtTDP-43 with 18E after indicated treatments. Scale bar: 50 μm. **f** Mixing score changes when comparing the mixing of wild-type TDP-43 with either 18E or 18A mutant under indicated treatment. The mixing scores were normalized to 1 under control conditions (DMSO) to help detecting a putative differential mixing. Changes in the normalized mixing score are represented for 13 kinase and 2 phosphatase inhibitors selected from the primary screen. Statistical significance of the bidirectionality was probed with an ANOVA test. Error bars indicate SEM and the asterisks indicate statistical significance with *p < 0.05, **p < 0.01, ***p < 0.001, ns non-significant, as measured by ANOVA test from *n*  =  4 wells. For each condition, the mean mixing score was measured in 4 wells (96-well plates). Kinase inhibitors (in red). Phosphatase inhibitor (in blue). **g** IC_50_ curves of the mixing score obtained from the analysis of 4 wells per condition. Incubation time: 4 h. The IC_50_ values are reported for 4 compounds.

To identify inhibitors of kinases or phosphatases that modify the phosphorylation-dependent TDP-43 assembly, we chose to investigate the mixing of wild-type TDP-43 with a hyper-phosphomimetic mutant having the highest number of serine residues mutated into glutamic acid (18E) to cover the maximum range of phosphorylation events. To evaluate the robustness of the MT bench assay, we measured a statistical indicator, the *Z*’ factor, in a 96-well plate^65^. When positive and negative controls were set as the mixing between wild-type TDP-43/18E and 18E/18A, respectively, we obtained a *Z*’ factor of 0.57 in our assay (Fig. 5a). This value demonstrates that our assay is suitable for screening molecules in a robust manner with *Z*’ > 0.5^66^ (see the “Methods” section). However, when we used wild-type TDP-43/wild-type TDP-43 as a control instead of 18E/18A, we obtained a *Z*’ factor of −0.80 (Supplementary Fig. 10a). This result indicates that the detection scheme is more sensitive to compounds that decrease the mixing score of wild-type TDP-43 with 18E, likely kinase inhibitors than those that increase the mixing score, likely phosphatase inhibitors. Using 18A rather than 18E as a mixing partner with wild-type TDP-43 may be a better option to detect potent phosphatase inhibitors with higher sensitivity.

A large-scale primary screen was then performed in HeLa cells with a commercially available library, including 98 kinase and 36 phosphatase inhibitors at a compound concentration of 10 µM after a 4-h incubation period (Tables S9, S10). A short incubation time was preferred to limit toxicity but should be sufficient to induce changes in the mixing of wild-type TDP-43 with its phosphomimetic mutants. The results indicate that most of the kinase and phosphatase inhibitors do not significantly impact the mixing score (*p* > 0.05, ANOVA test with quadruplicates). Only 14 kinase inhibitors and 2 phosphatase inhibitors significantly modify the mixing of wild-type TDP-43 with the hyper-phosphomimetic mutant (18E) (Fig. 5b–e). Importantly, kinase inhibitors tend to promote de-mixing with the hyper-phosphomimetic mutant, while phosphatase inhibitors promote mixing (Fig. 5c–e, *p* < 0.01 for both kinase and phosphatase inhibitor versus control). A differential behavior is expected if the kinase/phosphatase inhibitors respectively decrease/increase the number of phosphorylated serine residues in the C-terminal region or if, on average, kinase and phosphatase inhibitors interfere differently with TDP-43 self-assemblies mediated by the C-terminal domain. Notably, IC261, a kinase inhibitor, was discarded due to a significant microtubule destabilization at 10 µM (Supplementary Fig. 10c). Staurosporine, hypericin, and 9,10-Phenanthrenequinone induced significant morphological cellular changes at 10 µM, indicating that their actions may be at least partly nonspecific (Supplementary Fig. 10c). Additionally, among all the selected kinase inhibitors, hypericin unexpectedly decreased the mixing of wild-type TDP-43 with itself, which is unexpected unless changes in cell morphology biased the measurement of the mixing/de-mixing score (Supplementary Fig. 10b).

After the primary screen, we studied in detail 13 out of 14 selected kinases (IC261 was discarded, as indicated above) and the 2 phosphatase inhibitors. We then compared, in two different secondary screens, whether hypo- or hyper-phosphomimetic mutants (18A or 18E) differentially mixed with wild-type TDP-43 (Fig. 5f). In the case of kinase inhibitors, we observed a better mixing of wild-type TDP-43 with hypo-phosphomimetic than hyper-phosphomimetic mutants (4 kinase inhibitors with significant differences). In contrast, with the two selected phosphatase inhibitors, the mixing of wild-type TDP-43 was significantly higher with the hyper-phosphomimetic mutant (Fig. 5f). Such a differential pattern suggests that the selected kinase inhibitors directly decrease the number of phosphorylated residues in the C-terminus or indirectly interfere with the C-terminus of TDP-43 to better mix wild-type TDP-43 with TDP-43 hypo- than hyper-phosphomimetic mutant, while the trend is opposite in the case of the selected phosphatase inhibitors.

At 1 µM and again with a short incubation time (4 h), only 5 kinase inhibitors still significantly decreased the mixing of 18E with wild-type TDP-43: C16, Ro 31-8220 mesylate, 5-Iodotubercidin, Staurosporine, and Hypericin (Supplementary Fig. 10d). We also measured the IC_50_ values of 4 kinase inhibitors with the MT bench assay (Fig. 5g). IC_50_ values ranged from 533 to 1279 nM for three compounds: 5-Iodotubercidin, C16, and Ro 31-8220 mesylate. JNJ-7706621 is only active at high concentrations with an IC_50_ value of ~2.1 µM.

### In contrast to phosphatase inhibitors, selected kinase inhibitors impaired TDP-43 nuclear retention

To analyze whether the selected molecules interfere with TDP-43 subcellular distribution, we considered 11 kinase inhibitors in detail (ML-9 and BML-259 were no longer studied due to their weak effect on TDP-43 mixing compared to other kinase inhibitors (Fig. 5c)) and 2 phosphatase inhibitors.

**Fig. 6:**
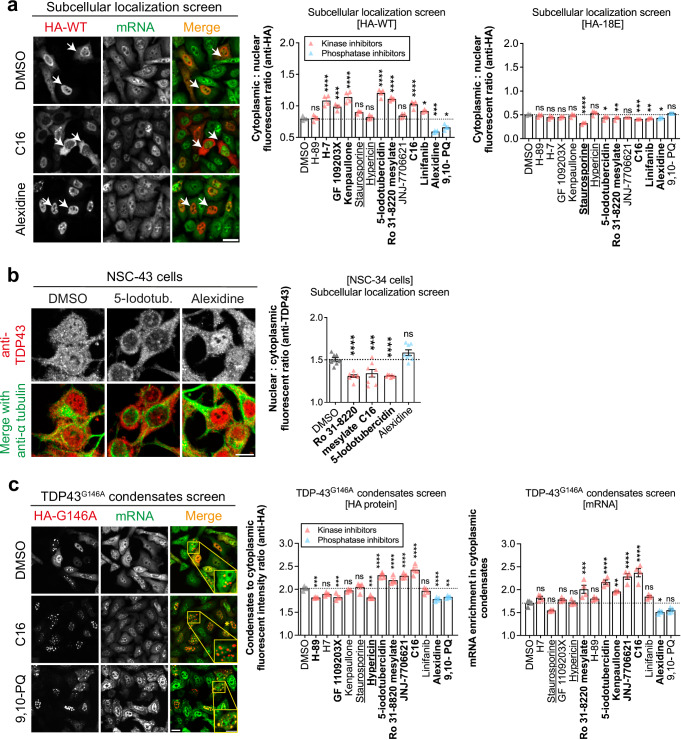
Selected kinase inhibitors antagonize the nuclear retention of TDP-43 in contrast with phosphatase inhibitors. **a** Left panel: Subcellular distribution of HA-tagged wild type TDP-43 in HeLa cells after treatment with the indicated compounds during 4 h (10 µM). Poly(A) mRNA in green (poly-dT probe) and anti-HA in red. Arrows indicate representative cells. Scale bar: 50 μm. Right panel: Automatic measurement of the cytoplasmic:nuclear ratio of HA-tagged TDP-43 under indicated conditions. Each data point represents the mean ratio value obtained from a single well in a 96-well plate. As control, the cytoplasmic:nuclear ratio of the hyper-phosphomimetic mutant was measured under the same condition. Error bars indicate SEM and the asterisks indicate statistical significance with *p < 0.05, **p < 0.01, ***p < 0.001, ****p < 0.0001, ns non-significant as measured by ANOVA test from *n* = 4 wells. **b** Left panel: Subcellular distribution of HA-tagged wild type TDP-43 in undifferentiated mouse motor neuron-like hybrid cell line, NCS-34 cells, after treatment with the indicated compounds for 4 h (3 µM). Poly(A) mRNA in green; anti-HA in red. Scale bar: 50 μm. Right panel: Automatic quantification of the nuclear:cytoplasmic ratio of HA-tagged TDP-43 under indicated conditions. Each data point represents the mean ratio value obtained from a single well. Error bars indicate SEM and the asterisks indicate statistical significance with ***p < 0.001, ****p < 0.0001, ns non-significant as measured by ANOVA test from *n* = 8 wells. **c** Left panel: Recruitment of HA-tagged G146A mutant in cytoplasmic condensates in HeLa cells treated with the indicated compounds during 4 h (10 µM). Anti-HA in red. mRNA in green. Scale bar: 50 μm. Zoom scale bar: 10 μm. Right panel: Automatic measurement of the enrichment of HA-tagged G146A or mRNA in cytoplasmic condensates versus cytosol under indicated conditions. Error bars indicate SEM and the asterisks indicate statistical significance with *p < 0.05, **p < 0.01, ***p < 0.001, ****p < 0.0001, ns non-significant as measured by ANOVA test from *n* = 4 wells.

After 4 h of incubation, strikingly, 7 kinase inhibitors, including the 5 kinase inhibitors still efficient at a 1 µM concentration, significantly modified the subcellular distribution of wild-type HA-TDP-43 expressed in the HeLa cells by promoting the cytoplasmic accumulation of TDP-43. The remaining kinase inhibitors were passive (Fig. 6a). In contrast, the 2 phosphatase inhibitors increased TDP-43 nuclear level. As a control, we observed that the 7 active kinase inhibitors that increased TDP-43 cytoplasmic level did not affect the subcellular distribution of the TDP-43 hyper-phosphomimetic mutant (18E) (Fig. 6a). We repeated this experiment in a motor neuron cell line (NSC-34) and probe the subcellular distribution of endogenous TDP-43 with an anti-TDP-43 antibody. We also used a lower concentration (3 µM for 4 h) to prevent cell toxicity. The three selected kinase inhibitors still efficient at 1 µM in HeLa cells (Supplementary Fig. 10d), Ro 31-8220 mesylate, 5-Iodotubercidin, and C16, significantly decreased the nuclear location of endogenous TDP-43 in NCS-34 cells (Fig. 6b). On the other hand, Alexidine, one of the two phosphatase inhibitors selected tended to increase TDP-43 nuclear retention. Staurosporine, hypericin, and, 9,10-Phenanthrenequinone, have to be considered with caution for this analysis since they significantly affected cell morphology (Supplementary Fig. 10c, d). Interestingly, the kinase and phosphatase inhibitors that we selected do not differentially affect the subcellular localization of FUS, another RNA-binding protein used as control, in contrast with TDP-43 (Supplementary Fig. 11). Indeed, these selected compounds, regardless of whether they are kinase or phosphatase inhibitors, moderately promote the cytoplasmic localization of FUS protein.

These results demonstrate that the kinase inhibitors promoting the de-mixing of wild-type TDP-43 with the hyper-phosphomimetic mutant tend to increase the cytoplasmic level of TDP-43. In contrast, the two phosphatase inhibitors were either passive or negatively regulated TDP-43 cytoplasmic levels. Only three kinase inhibitors, Ro 31-8220 mesylate, 5-Iodotubercidin, and C16, were validated in all the screens (active at 1 µM in MT bench assay, translocation of HA-wild-type TDP-43 in HeLa cells, and translocation of endogenous TDP-43 in NSC-34 cells). However, since kinase inhibitors have broad target spectra, it is possible that alterations in the mixing of wild-type TDP-43 with hyper-phosphomimetics may result from indirect effects that impact TDP-43 C-terminus self-assembly. In addition, from the list of selected hits, it is not possible to draw conclusions about the involvement of specific kinases in the regulation of TDP-43 self-association via its C-terminus in the cytoplasm.

### Kinase inhibitors promote the formation of aggregates in an mRNA-binding deficient TDP-43 mutant

We investigated whether the selected molecules interfere with the recruitment of TDP-43 into stress granules. We performed this screen in HeLa cells expressing wild-type HA-TDP-43 and G3BP1-GFP to form stress granules in the absence of additional oxidative stress (Supplementary Fig. 12). Of the 11 kinases and 2 phosphatase inhibitors tested, we observed a poor correlation between the recruitment of TDP-43 into stress granules and changes in the mixing of wild-type TDP-43 with the hyper-phosphomimetic mutant in the cytoplasm (Supplementary Fig. 12, Fig. 5f). Moreover, we observed unexpected variations in the recruitment of TDP-43 hyper-phosphomimetic mutant in stress granules. Therefore, the selected kinase/phosphatases inhibitors could alter the recruitment of TDP-43 into stress granules independently of mechanisms linked to TDP-43 self-assembly controlled by the phosphorylation state of its C-terminal domain in the cytoplasm. Consistent with the limited correlation between TDP-43 recruitment in stress granules and TDP-43 self-assembly orchestrated by the C-terminus, we found no overlap between the kinase inhibitors and those identified in a previous screen based on the enrichment of TDP-43 in stress granules^54^ (Supplementary Fig. 13a).

Next, we sought to investigate whether the selected molecules interfere with TDP-43 self-assembly by using a TDP-43 mutant, G146A, which strongly impairs the recruitment of TDP-43 in mRNA-rich stress granules. G146 residue is located in an unstructured loop of RRM1, which controls the cooperative binding of TDP-43 to GU-rich RNA repeats^7^. When G146A is expressed in HeLa cells, it forms G146A-rich cytoplasmic condensates that are poorly enriched in mRNA, as previously reported^7^. We measured the recruitment of G146A into condensates and their relative enrichment in mRNAs in HeLa cells treated with the selected compounds for 4 h. The two phosphatase inhibitors clearly inhibited G146A condensation, whereas 4 out of the 11 tested kinase inhibitors, including the 3 hits identified from the MT bench assay were still efficient at 1 µM (Ro 31-8220 mesylate, 5-Iodotubercidin and C16) (Fig. 6c, Supplementary Fig. 10d), increased the recruitment of TDP-43 in aggregates, indicating a negative impact on G146A solubility (Fig. 6c). Interestingly, Ro 31-8220 mesylate, 5-Iodotubercidin, and C16 also increased the content mRNA in G146A-rich condensates, unlike the 2 phosphatase inhibitors (Fig. 6c). This result is in keeping with the stronger recruitment of mRNAs by hypo- than hyper-phosphomimetic mutants detected on microtubules (Fig. 4b).

We also analyzed whether the selected compounds could interfere with the splicing events controlled by TDP-43 (Supplementary Fig. 13b). Using the reporter of *CFTR* exon 9 excisions controlled by TDP-43, we found that some compounds affected the skipping of exon 9, but we failed to find a clear trend with the mixing score. Notably, Ro 31-8220 mesylate but not 5-Iodotubercidin and C16 affected the skipping of exon 9. As the selected compounds were screened for their ability to interfere with cytoplasmic TDP-43 self-assemblies, a limited correlation with mRNA splicing taking place in the nucleus may be expected.

In summary, in contrast to translocation assays, neither the stress granule nor splicing assays revealed a clear correlation with the MT bench assays (Figs. 5f and 6a). However, we notice that kinase inhibitors, validated at low concentration (1 µM), and the two phosphatase inhibitors promoted and inhibited cytoplasmic condensation of the G146A mutant, respectively (Fig. 6c).

### Selected kinase inhibitors prevent the import of cytoplasmic TDP-43 into the nucleus

**Fig. 7:**
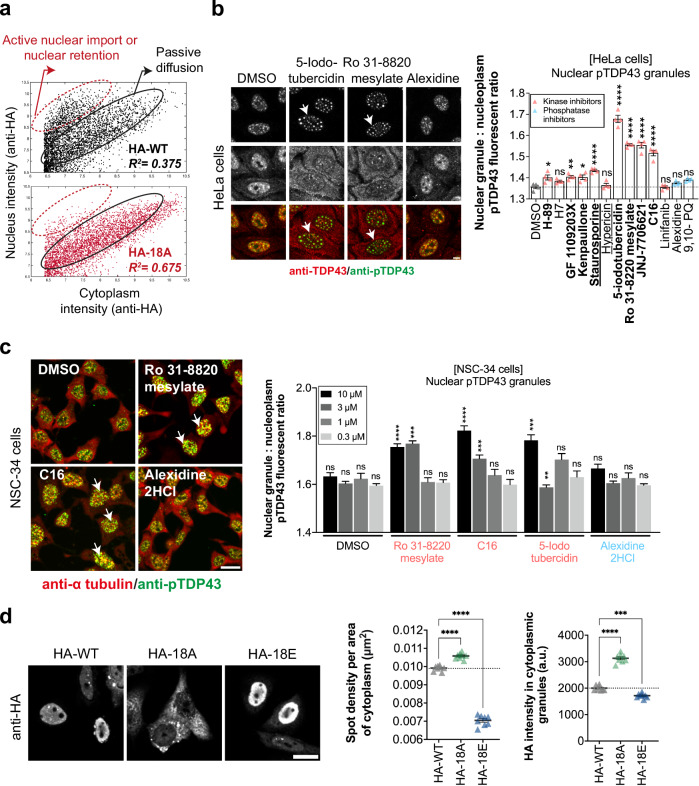
Kinase inhibitors and the hypo-phosphomimetic mutations (18A) impaired TDP-43 nuclear retention because of cytoplasmic TDP-43 self-assembly. **a** Scatter plot of subcellular distribution of HA-tagged wild type and hypo-phosphomimetic TDP-43. A linear regression with a high *R*² (0.675) shows that the level of cytoplasmic and nuclear hypo-phosphomimetic mutant increase linearly, as expected for passive diffusion. On the other hand, in HeLa cells expressing wild-type TDP-43, we observed a fraction of the cells with increased TDP-43 nuclear levels. Each dot represents a single cell. The outlines were done by hand to help the reader. **b** Left panel: Representative images of HeLa cells treated with the indicated compounds during 4 h (10 µM). Red, endogenous TDP-43. Green, anti-phospho TDP-43 (Ser 409/410). Arrows indicate an increased brightness of anti-phospho-TDP-43 positive nuclear granules. Arrows show representative nuclear granules. Scale bar: 20 µm. Right panel: Quantification of fluorescence ratio of anti-phospho TDP-43 inside versus outside nuclear granules. Only nuclei were selected for this analysis. Error bars indicate SEM and the asterisks indicate statistical significance with *p < 0.05, **p < 0.01, ****p < 0.0001, ns. non-significant as measured by ANOVA test from *n*  =  4 wells. **c** Same as (**b**) with mouse motor neuron-like hybrid cell line NSC-34. Left panel: Representative images of NSC-34 cells. Red, tubulin. Green, anti-phospho TDP-43. Arrows show representative nuclear granules. Scale bar: 30 µm. Right panel: statistical analysis. The effect was assessed for four concentrations of compounds. Error bars indicate SEM and the asterisks indicate statistical significance with **p < 0.01, ***p < 0.001, ****p < 0.0001, ns non-significant as measured by ANOVA test from *n*  =  4 wells. **d** Left panel: Representative images of HeLa cells expressing HA-tagged TDP-43 or indicated mutant. Arrows indicate representative cytoplasmic TDP-43-rich granules. Scale bar: 30 μm. Zoom scale bar: 20 μm. Right panel: Quantification of the anti-HA intensity in detected cytoplasmic spots and the density of spots per cytoplasm area. Each dot represents the mean value obtained over the selected cells in a single well. Error bars indicate SEM and the asterisks indicate statistical significance with ***p < 0.001, ****p < 0.0001, ns non-significant as measured by ANOVA test from *n*  =  8 wells.

A correlation has been established between compounds that reduce the mixing of wild-type TDP-43 with the hyper-phosphomimetic mutant (18E) in the cytoplasm and an increased presence of wild-type TDP-43 in the cytoplasm, while the opposite trend is observed with phosphatase inhibitors (Figs. 5f and 6a). To explain the phenomena behind this correlation, we examined the nuclear levels of wild-type TDP-43 and TDP-43 hypo- and -hyper phosphomimetic mutants at the single-cell level compared to their respective levels of expression in HeLa cells. We observed a population of cells displaying a marked nuclear location for both wild-type TDP-43 and the hyper-phosphomimetic mutant, 18E (Supplementary Fig. 14a). This behavior is consistent with an active nuclear import of TDP-43^49,67^ and/or a nuclear retention^48^ based on the formation of TDP-43 higher-order assemblies in the nucleus (Fig. 7a). In contrast, the hypo-phosphomimetic mutant (18A) displays a linear relationship between nuclear and cytoplasmic levels, with a higher *R*² value compared to wild-type TDP-43 or 18E, which is the distribution expected for passive diffusion. The population of cells displaying a marked TDP-43 nuclear location while having a limited cytoplasmic location has decreased as if active nuclear import has been impaired for 18A (Fig. 7a, Supplementary Fig. 14a).

Interestingly, the same pattern was observed in HeLa cells expressing HA-TDP-43 treated with our three kinase inhibitor hits, Ro 31-8220 mesylate, 5-Iodotubercidin, and C16 (Supplementary Fig. 14a). Based on these results, we investigated whether its nuclear retention is promoted after cell treatments with kinase inhibitors. We first noticed the presence of granular nuclear structures enriched in phosphorylated TDP-43 in HeLa cells or NSC-34 cells (anti-phospho TDP-43, serine residues 409/401) (Fig. 7b, c) that may be associated with cellular stress^68^. These nuclear granular-like structures may represent sites in which phosphorylated TDP-43 but also wild-type TDP-43 can be sequestered in the nucleus. Indeed, HA-TDP-43, HA-18E but not HA-18A were detected in the anti-phospho TDP-43-rich nuclear granules (Fig. 2d). However, upon expression of hyper- but not hypo-phosphomimetic mutants, we observed that the recruitment of TDP-43 in these nuclear granules did not increase (Supplementary Fig. 14b). Therefore, the expression of hyper-phosphomimetic TDP-43 mutants did not promote the assembly of the phospho-TDP-43-rich granular structures. More importantly, we also observed that the selected kinase inhibitors, which markedly increase TDP-43 cytoplasmic levels, promoted the formation of phospho-TDP-43 nuclear granules in both HeLa and NCS-34 cells (Fig. 7b, c). Thus, the increased cytoplasmic TDP-43 level upon cell exposure to the selected kinase inhibitors is not due to the dissociation of phosphorylated TDP-43 nuclear granules.

Then, we investigated whether the hypo-phosphorylated TDP-43 could be more retained in the cytoplasm than the hyper-phosphorylated mutant. In a previous study^43^, it was reported that cytoplasmic de-mixing of TDP-43 inhibits its nuclear transport, leading to the depletion of nuclear TDP-43. We then examined whether TDP-43 self-assemblies in the cytoplasm increase when cells express a hypo-phosphomimetic mutant. While most cells showed a homogenous distribution for TDP-43 hyper-phosphomimetic mutant (18E), we observed an increase in the occurrence of TDP-43-rich granules in the cytoplasm of cells expressing the TDP-43 hyper-phosphomimetic mutant. Using the same window of TDP-43 expression in the cytoplasm, we also found that the enrichment of TDP-43 in the detected granules was highest with the hypo-phosphomimetic mutant (18A) and lowest with the hyper-phosphomimetic mutant (18E) (Fig. 7d). In addition, denser compartments were generated along microtubules by the hypo-phosphomimetic mutant compared to the hyper-phosphomimetic mutant (Supplementary Fig. 3a).

## Discussion

**Fig. 8:**
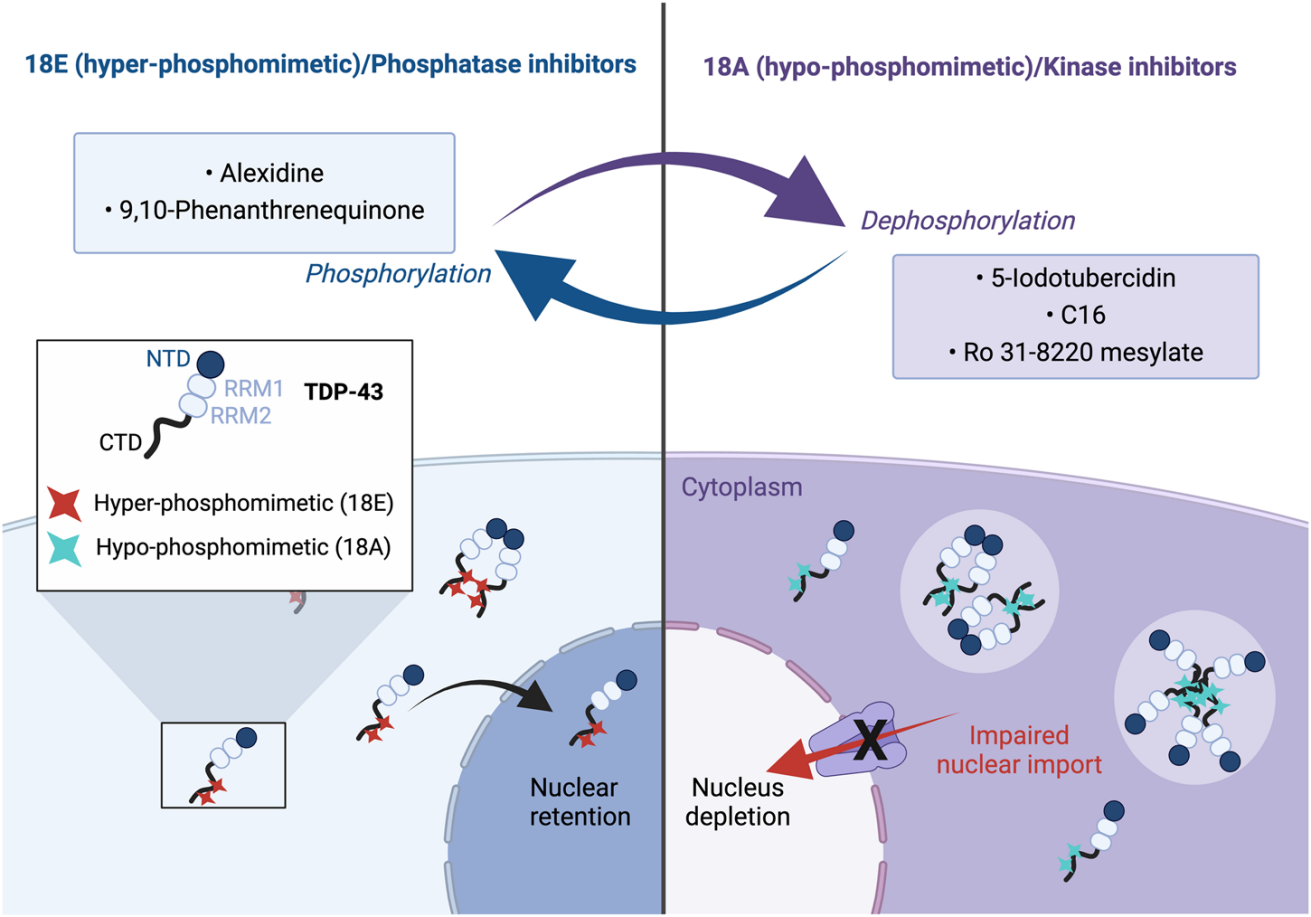
Schematic view of the link between phosphorylation of the C-terminal domain of TDP-43 and its sub-cellular location in cells. According to our results, the dephosphorylated state of TDP-43 promotes its cytoplasmic retention with cytoplasmic condensates as observed in the case of 18A mutant or in the presence of protein kinase inhibitors. Indeed, the hypo-phosphorylation of TDP-43 in the cytoplasm positively regulates protein-protein interaction, which antagonizes the import of cytoplasmic TDP-43 to the nucleus leading to TDP-43 nuclear depletion and its retention in the cytoplasm. In contrast, as observed for 18E mutant as well as in the presence of protein phosphatase inhibitors, phosphorylated TDP-43 is enriched in the nucleus.

Deciphering the mechanisms that regulate TDP-43 cytoplasmic/nuclear levels is critical to defining therapeutic strategies since the presence of TDP-43 in cytoplasmic inclusions with less TDP-43 in the nucleus is a pathological hallmark of ALS and FTLD^2^. In previous studies, passive diffusion of TDP-43 through nuclear pores associated with an active nuclear import has been proposed as the main determinant of TDP-43 sub-cellular distribution^49,67^. According to this model, an increased TDP-43 sequestration in nuclear structures/compartments promotes TDP-43 nuclear retention^48,69^. Alternatively, an increased occurrence of TDP-43 self-assemblies in the cytoplasm has also been reported as a factor that increases the cytoplasmic location of TDP-43 by reducing its nuclear import^43^ (Fig. 8). Consistent with the latter point, removing the C-terminus domain of TDP-43 promotes its cytoplasmic location^50^. The TDP-43 cytoplasmic self-assembly hypothesis also makes sense because hypo-phosphorylation of the TDP-43 C-terminal promotes the formation of liquid droplets with reduced dynamics in vitro^28^ (Fig. 8 and Supplementary Fig. 3a). We thus considered whether the sub-cellular location of TDP-43 can be differentially regulated by kinase and phosphatase inhibitors that alter the phosphorylation state of the self-adhesive C-terminal domain of TDP-43 in the cytoplasmic.

Here, we used the Microtubule Bench method to select kinase and phosphatase inhibitors that alter the phosphorylation-dependent self-assembly of TDP-43 mediated by the C-terminus domain in the cytoplasm (Fig. 1b). Having identified kinase and phosphatase hits (Fig. 5c), we found that both TDP-43 hypo-phosphorylation mimetics and, consistently, the three kinase inhibitor hits, Ro 31-8220 mesylate, 5-Iodotubercidin, and C16, negatively regulate the nuclear retention of TDP-43. In contrast, the hyper-phosphomimetic mutant and the selected phosphatase inhibitors increase TDP-43 nuclear levels (Figs. 3a and 6a). Therefore, there is a clear negative correlation between the strength of cytoplasmic TDP-43 self-assembly (mixing score on microtubules) and the efficiency of TDP-43 nuclear retention. In addition, when we used G146A, a TDP-43 mutant that no longer cooperatively binds to GU-rich sequence, to form cytoplasmic TDP-43 condensates, we found that the phosphatase inhibitors oppose the sequestration of G146A into cytoplasmic condensates (Fig. 6c). On the other hand, the three kinase inhibitor hits increase the sequestration of G146A mutant into cytoplasmic condensates (Fig. 6c).

Of note, phosphorylated TDP-43 recognized by anti-phospho TDP-43 antibodies (S409/410) is generally associated with a cytoplasmic location of TDP-43 and a positive regulation of TDP-43 self-assembly. These observations were often related to the aggregation of TDP-43^30,70–72^, which occurs at the late stages of ALS or FTLD^73^. Therefore, cytoplasmic TDP-43 phosphorylation in neurons affected by these diseases could also occur downstream of TDP-43 aggregation and truncation^39^.

In addition to analyzing sub-cellular location of TDP-43, the results obtained with a CFTR exon 9 mini-gene reporter indicate a possible role of TDP-43 phosphorylation in splicing regulation (Fig. 3c). However, there is no correlation between changes in cytoplasmic self-assembly of TDP-43 and the inclusion of exon 9 of the *CFTR* gene after treatment with the selected kinase and phosphatase inhibitors (Supplementary Fig. 13b). TDP-43 control over nuclear splicing events may not be regulated by the same kinases, which makes sense as splicing takes place in the nucleus and not in the cytoplasm.

Another point is the recruitment of TDP-43 mutants in stress granules which can be impaired by TDP-43 hyper-phosphorylation of its C-terminus^28^. Here we clarified that stress granules obtained after G3BP1 overexpression or after mild oxidative stress (300 µM H_2_O_2_) do recruit the hyper-phosphomimetic mutants (Fig. 4a, Supplementary Fig. 7b–d). The non-recruitment of the hyper-phosphomimetic mutants in stress granules occurs only after acute oxidative stress, such as arsenite treatment or elevated H_2_O_2_ concentration (1 mM). Acute oxidative stress promotes intermolecular RRM interactions^41,42^, which strongly inhibit the binding of the hyper-phosphomimetic mutant TDP-43 to mRNA, but to a lesser extent for the hypo-phosphomimetic mutants (Fig. 4b). Since stress granules are mRNA-rich compartments, an impaired affinity of TDP-43 for cytoplasmic mRNA may explain the non-recruitment of the hyper-phosphomimetic mutant in stress granules under acute oxidative stress (Fig. 4b, Supplementary Fig. 7b–d). Whether phosphorylation of the TDP-43 C-terminus controls the binding of TDP-43 to mRNA under acute stress and whether this behavior is only observed in artificial phosphomimetic mutants remain open questions.

In summary, TDP-43 self-assembly in the cytoplasm, promoted by the hypo-phosphorylation of the C-terminal domains, antagonizes TDP-43 nuclear import. As already proposed^43^, with the objective of defining a therapeutic strategy, decreasing TDP-43 C-terminus self-assembly in the cytoplasm is a priori relevant to promote the nuclear localization of TDP-43 and limit TDP-43 cytoplasmic aggregation. Given that many pathological mutations occur in the C-terminal domain, it is important to investigate whether phosphorylation or dephosphorylation events can correct the deregulation in TDP-43 self-assembly and TDP-43 functions in mRNA processing, especially splicing. Post-translational modifications of TDP-43 can also interfere with mitochondrial functions^59^ since TDP-43 phosphorylation could increase the presence of TDP-43 in mitochondria (Supplementary Fig. 6), which in turn may increase TDP-43 neurotoxicity. Conversely, it is also important to investigate whether pathological mutations alter the phosphorylation status of the C-terminal domain. These are crucial issues that require further investigation in order to better understand the mechanism underlying TDP-43-related neurodegenerative diseases and to develop potential therapeutic strategies.

## Methods

### Plasmid preparation for MT bench experiments

Plasmids harboring the full-length TDP-43, HuR, and G3BP1 genes fused with RFP-MBD (microtubule-binding domain of Tau) and/or GFP-MBD were obtained previously^7,40^. Plasmids used for the MT bench technique are listed in Table S1. Constructed Hypo- and hyper-phosphomimetic mutations are located in the C-terminal domain of TDP43, and DNA sequence encoding for this part is located between *NdeI* and *AscI* restriction sites in the plasmid TDP43-RFP-MBD. Hypo- and hyper-phosphomimetic constructs were amplified by PCR using a synthetic DNA template bearing the desired mutations (purchased from Eurofins Genomics) and primers containing NdeI and AscI restriction sites. Then, they inserted into the plasmid TDP43-RFP-MBD digested with NdeI and AscI restriction enzymes. The introduced mutations were checked by DNA sequencing (Eurofins Genomics). All the DNA oligos used for constructing TDP43 hypo- and hyper-phosphomimetic mutants are indicated in Table S2.

### Cell culture and plasmid co-transfection for MT bench experiments

HeLa cells were obtained from ATCC and cultured at 37 °C, 5% CO_2_ in high-glucose DMEM medium (Dulbecco’s modified Eagle’s medium) supplemented with 10% fetal bovine serum (FBS), 2 mM l-glutamine, 1x MEM non-essential amino acids solution and 1% penicillin–streptomycin (GIBCO Life Technologies). Cells at confluence were plated at a density of 18,000 cells per well of 96-well plates and co-transfected with the indicated plasmids using Lipofectamine 2000 reagent (Invitrogen) as a vehicle. Co-transfected cells were incubated during 24 h at 37 °C in 5% of CO_2_. Cells were washed with PBS, then fixed with ice-cold methanol for 10 min at –20 °C and washed with PBS. Cells were further fixed with 4% paraformaldehyde (PFA) diluted in PBS for 10 min at RT. This double methanol/PFA fixation is performed to improve the microtubule structure visualization. After final washing with PBS, 96-well plates were ready for image acquisition with the Opera Phenix® Plus High-Content Screening (HCS) System.

### Image acquisition and mixing score (*R*-squared) measurement

All image acquisition was carried out with Opera Phenix® Plus High Content Screening System (PerkinElmer) in confocal mode on ×40 magnification. To assess sub-compartmentalization in the system with both proteins fused to GFP/RFP-MBD, the cell image analysis was performed using the PerkinElmer Harmony v4.8 software, and we have defined an analysis pipeline by applying several parameters defining spatial segregation of two proteins such as RFP/GFP fluorescence intensity in the spot, number of spots, width to length ratio of the spot and RFP/GFP fluorescence intensity in the cytoplasm. Briefly, we selected the population of interest, i.e. the co-transfected cells harboring both red and green cells. Nuclei, cytoplasm, and spots of proteins fused to GFP/RFP-MBD along the microtubule network were automatically detected. we identified clusters of microtubules (spots) along the microtubules of the cells, and we applied thresholds to filter them. The spots were selected according to their area (30 px^2^ < spot area <200 px^2^) and the width-to-length spot ratio (<0.2) in order to remove all non-tubular spots. In each selected “microtubule” spot, the intensity of RFP and GFP was measured as well as in the surrounding cytoplasm. The mixing score (*R*-squared) was obtained by plotting the mean intensity values (*I*_RFP-TDP43_/*I*_GFP-TDP43_) having previously subtracted the RFP and GFP intensities in the cytoplasm to eliminate the background. A linear regression has been used to automatically determine *R*^2^ values.

### Plasmid preparation for cellular functional bioassay

Hypo- and hyper-phosphomimetic constructs harboring HA-tag (YPYDVPDYA) were amplified by PCR using a synthetic DNA template bearing the desired mutations (purchased from Eurofins Genomics) and primers containing NdeI and AscI restriction sites. Plasmids harboring the full-length TDP-43, HuR, and G3BP1 genes fused with HA-tag were obtained previously^7^. In order to construct the plasmids encoding the full-length TDP43 Hypo- and hyper-phosphomimetic fused to HA-tag, similar procedure is described above for plasmids used in MT bentch assay. The corresponding insert was amplified by PCR using primers containing NdeI and XhoI restriction sites and inserted into HA-TDP43-pcDNA3.1 plasmid digested with *NdeI* and *XhoI* restriction sites^7^.

### Phosphorylation status

After transfection with the indicated plasmid and fixation with 4% PFA, IF was realized by using anti-HA (1:1000, Santa Cruz Biotechnology) and anti-phospho-TDP43^Ser409/410^ (1:3000, PAB Proteintech) primary antibodies diluted in blocking buffer. The phosphorylation level was quantified by measuring the pTDP43:anti-HA fluorescence ratio.

### Subcellular localization assay

HeLa cells were transfected for 24 h with indicated HA-tagged phosphomimetic mutants or 48 h post-transfection followed by DMSO or 300 μM actinomycin D (ActD) treatment during 1 h 30 at 37 °C. After treatment, cells were fixed with 4% PFA for 30 min 37 °C. The staining was performed using anti-HA (1:1000, Santa Cruz Biotechnology) primary antibodies. mRNA was visualized by in situ hybridization with Cyn3 or Cyn2-labelled poly(dT) probes. Quantifications were performed with HCS confocal mode. The HCS software was used to automatically detect the fluorescence intensity of HA in cells. Cytoplasm was revealed by the mRNA fluorescence. We measured the cytoplasmic: nuclear fluorescence ratio by using the anti-HA fluorescence signal.

### Splicing reporter analysis

To generate stable HEK-293T cell lines for splicing reporter analysis, 50,000 HEK-293T cells from ATCC were plated in six-well plates using standard conditions (DMEM, 10% FBS, penicillin–streptomycin). The indicated lentiviral plasmid targeting *TARDBP* (AM21) or negative control TRS-NS (see Table S5) were packaged using second-generation packaging plasmids (pGIPZ, pMD2.G, psPAX2) (Addgene) and transfected into HEK-293T target cells with Effectene transfection reagent (Qiagen) to produce lentiviral particles for transduction. Viral supernatants were collected at 48 h post-transfection, pooled, and filtered through non-binding 0.45 μm syringe filters and 40 μL was used to transduce HEK-293T cells with 8 μg/μL polybrene (Sigma-Aldrich). After 24 h, the virus-containing medium was removed and replaced with a selection medium containing Puromycin 8 μg/μL (Sigma-Aldrich). After 7 days of selection, cells were stored at −80 °C. Stable inhibition of TDP43 was verified by immunofluorescence and western blot and stable HEK-293T line AM21 was selected to use for splicing reporter analysis experiments. The AM21 shRNA targets a coding sequence of *TARDBP* gene in the 3’ part. Therefore, to avoid off-target effects, we synthesized oligonucleotides in order to introduce mismatch with silent mutations without altering the function of the TDP43 protein. Table S6 shows, for each plasmid, the corresponding primers used to introduce the mismatch mutations. TDP43 functional activity was assessed using a transfection CFTR splicing reporter from *Rajat Rohatgi* lab (pHBS956 IBB-GFP-mCherry3E; Plasmid #107859; Addgene). The corresponding HA plasmids were co-transfected with *CFTR* splicing reporter into shRNA stable cell line-HEK293T using Lipofectamine 2000 reagent (Invitrogen) following the standard manufacturer’s protocols. After 24 h, cells were fixed and stained with anti-HA antibody (1:1000, Santa Cruz Biotechnology). Splicing activity was then evaluated by IF in the nucleus using HCS imaging. The fluorescence intensity of GFP and mCherry in cells was detected in the nucleus, and we measured the mean intensity ratio of mCherry:GFP for each condition.

### Stress granules assay

HeLa cells were co-transfected with corresponding HA plasmid for indicated times.

For stress granules induced with Arsenite, cells were treated with DMSO or 300 μM Arsenite for 1 h at 37 °C. After treatment, cells were washed with PBS and fixed with 4% PFA for 30 min at 37 °C. The staining was performed using anti-HA (1:1000, Santa Cruz Biotechnology) primary antibody (Tables S7, S8). To visualize mRNA in HeLa cells, mRNA in situ hybridization was performed. Briefly, cells were fixed with 4% PFA as explained above. Cells were then incubated with ethanol 70% for 10 min at RT and then with 1 M Tris–HCl pH 8.0 solution for 5 min. Poly-dT oligonucleotide coupled with Cyn-2 1 μg/μL (Molecular Probed Life Tech) in the hybridization buffer (0.005% BSA, 1 mg/mL yeast RNA, 10% dextran sulfate, 25% formamide in 2X SSC buffer) was then used to reveal mRNAs. DAPI (0.66 mg/mL) was used to visualize nuclei. Quantifications were performed with HCS imaging in confocal mode. The HCS software was used to assess the anti-HA and (or) fluorescence intensity in SGs and in the cytoplasm for background (Supplementary Fig. 7). The type of measurement is indicated in each figure legend. For Fig. 4a, we measured the slope of the enrichment of HA-tagged protein (SG/Cytoplasm) versus the enrichment of G3BP1-GFP (SG/Cytoplasm). A zero slope means that the HA-tagged protein is not recruited in SGs (Supplementary Fig. 15).

For stress granules formation with hydrogen peroxide (H_2_O_2_), cells were first pre-treated with puromycin (2.5 μg/mL) for 20 min and then with H_2_0_2_ (300 μM or 1 mM) for 30 min at 37 °C. Cells were fixed as mentioned above and subjected to IF by using anti-HA antibody (1:1000, Santa Cruz Biotechnology). mRNA was visualized by in situ hybridization as described above.

For the Bait/Prey assay, cells were co-transfected with the corresponding plasmid and were treated with DMSO or Arsenite (300 μM) for 1 h at 37 °C. After 4% PFA fixation, the staining was performed using an anti-HA antibody (1:1000, Santa Cruz Biotechnology).

To investigate TDP43-GFP and HA protein recruitment in SGs following arsenite stress, cells were co-transfected with the corresponding plasmid and were treated with 300 μM Arsenite during 1 h at 37 °C. The staining was done with anti-HA antibody (1:1000, Santa Cruz Biotechnology). mRNA was detected by in situ hybridization as above-mentioned. Quantification of HA and TDP43-GFP protein relative recruitment in SGs versus cytoplasm was performed.

### RNA interaction assay

Cells were transfected with a single bait plasmid bearing point mutations (18E-RFP-MBD, 18A-RFP-MBD, or the wild-type TDP43-RFP-MBD plasmid) and treated with DMSO or Arsenite (300 µM) during 1 h at 37 °C. After cell treatment, cells were fixed and mRNA in situ hybridization was performed to assess mRNAs in cells as mentioned above. The bait and mRNA fluorescence was detected automatically and the ratio *I*_bait_/*I*_mRNA_ was measured to evaluate the interaction of the bait protein with mRNA.

### Mitochondrial localization of TDP-43 assessment

After transfection with the indicated plasmid, localization of TDP-43 in mitochondria was assessed by using anti-TOM40 staining with the following measurement of HA-tagged TDP-43 mutants in the corresponding mitochondria region.

### Lactate dehydrogenase release assay

After transfection with the indicated plasmid, the cell cytotoxicity was measured using CytoTox-ONE™ homogenous membrane integrity assay (Promega). The mean LDH release from mock-transfected cells treated with lysis solution was used as the maximum cytotoxic effect.

### Cell viability assay

After transfection with the indicated plasmid, the viability of cells was then measured by MTT assay (Sigma-Aldrich) according to the manufacturer’s instructions. Absorbance at 570 nm was measured using a microplate reader and relative cell viability was calculated compared to mock-transfected cells.

### Compounds used in MT bench screen

We selected a phosphatase and kinase inhibitors libraries (ENZO) and others known phosphatase and kinase inhibitors (Tables S9, S10). Compounds were provided and prepared (10 mM in a 96-well plate) by Mcule company.

### Assay validation and hit screen

The assay was validated using the *Z*’ factor measure. For this, a series of negative (screening system) and positive controls (de-mixing) were measured using the MT bench assay. Briefly, HeLa cells were plated on 96-well plates (CellCarrier Ultra microplates, PerkinElmer) at a density of 18,000 cells per well. After overnight incubation, cells were co-transfected for each condition in 48 wells with the following couple of plasmids based on the optimization of the transfection conditions: 18E-RFP-MBD/wtTDP43-GFP-MBD for the screening system and the couple 18A-RFP-MBD/18E-GFP-MBD for the de-mixing control by using 0.4 μL lipofectamine 2000^TM^ reagent (Invitrogen). DNA concentrations used for this screen are 0.4 µg/0.1 µg per well for RFP-MBD/GFP-MBD plasmids, respectively, since GFP fusion is better expressed in HeLa cells. After 24 h co-transfection, cells were fixed with methanol/4% PFA. The plate was analyzed, and the *Z*’ factor was measured. To validate the degree of separation, the *Z*’ factor was determined using the formula:

$$Z^{\prime}\, {\rm {{factor}}}=1-\frac{\,3\left({\sigma }_{{{\rm {SS}}}}+{\sigma }_{{{\rm {DS}}}}\right)}{\left({\mu }_{{{\rm {SS}}}}-{\mu }_{{{\rm {DS}}}}\right)}$$

where ${{{\rm{\sigma }}}}_{{{\rm{SS}}}}$ and ${\sigma }_{{{\rm {DS}}}}$ are the standard deviations of the screening system (SS) and de-mixing system (DS), respectively, and ${\mu }_{{{\rm {SS}}}}$ and$\,{\mu }_{{{\rm {DS}}}}$ are the corresponding mean values. A *Z*’ factor between 0.5 and 1.0 indicates an excellent assay and statistically reliable separation between the screening system and de-mixing system. Using a BRAVO liquid handler (Agilent), HeLa cells were seeded and were co-transfected in quadruplicate with the screening system (wtTDP43-GFP-MBD/18E-RFP-MBD) or the de-mixing control (18A-RFP-MBD/18E-GFP-MBD) to performed quality control (*Z*’ factor measurements for each screen plate) with the same DNA concentrations as those used for assay validation. Cells were treated in quadruplicate with DMSO or drug compounds at desired concentrations. Only data at 10 μM will be shown. Then cells were exposed to compounds for 4 h at 37 °C. After compound treatment, cells were fixed. Nuclei were stained with 1 mg/mL DAPI. Image acquisition was performed automatically with the Opera Phenix® Plus High-Content Screening System. Details on image acquisition and statistical analysis are provided in Fig. 2a, b.

### Determination of IC_50_ of hit compounds

HeLa cells were co-transfected with wtTDP43-GFP-MBD/18E-RFP-MBD plasmids and treated with hit compounds. Ten concentrations of drugs were tested. Experiments were done in quadruplicates for each concentration. IC_50_ values were determined by plotting the % inhibition versus the log of the concentration of the inhibitor. The inhibitor concentrations used are: 39.063, 78.125, 156.250, 312.500, 625, 1250, 2500, 5000, 10000, and 20000 nM. After 4 h of incubation, cells were fixed, and the quantification was performed as described above.

### Cell-based bioassay screen

HeLa cells were transfected with the indicated plasmid and then treated with the 13 hit compounds (10 µM) for 4 h at 37 °C.

### Subcellular localization screen

After incubation of molecules, HeLa cells were washed and fixed as already mentioned above. mRNA was visualized by in situ hybridization. The staining was performed using an anti-HA primary antibody. The same experiment was carried out using the NSC-34 cell line. NSC-34 cells were purchased from ATCC and maintained in DMEM supplemented with 10% fetal bovine serum (FBS) and penicillin and streptomycin antibiotics (pen/strep). NSC-43 cells were seeded in 96-well plates and treated with DMSO or hit compounds. Immunostaining was performed with anti-TDP43 (1:1000, Proteintech) and anti-α-Tubulin (1:1000, Sigma-Aldrich) primary antibodies. Quantifications are already mentioned above in the subcellular localization assay section.

### Stress granule screen

After hit compounds treatment, the cells were fixed as mentioned above. The staining was performed using anti-HA primary antibody (1:1000, Santa Cruz Biotechnology).

### TDP43^G146A^ condensates screen

HeLa cells were transfected with the indicated plasmid (TDP43 G146A mutant) and then treated with hit compounds for 4 h at 37 °C. IF was performed with anti-HA antibody and mRNAs were stained by in situ hybridization as described above. We measured the aggregates to cytoplasmic fluorescence ratio by using anti-HA fluorescence and the mRNA enrichment in cytoplasmic aggregates by using the mRNA fluorescence assessment.

### Phosphorylation screen

After drug treatment, IF was performed with the following primary antibodies: anti-TDP43 (1:1000, ABnova) and anti-pTDP43 (1:3000, Proteintech). The same experiment was carried out with the NSC-34 cell line. NSC-34 cells were treated with the indicated compounds and IF was performed with anti-α-Tubulin (1:1000, Sigma-Aldrich) and anti-TDP43 (1:1000, Proteintech) primary antibodies. We quantified the fluorescence ratio of anti-pTDP43 inside versus outside nuclear granules by the nuclear granule:nucleoplasm pTDP43 fluorescence ratio.

### Splicing screen

HEK-293T stable lines were transfected with the indicated plasmid and *CFTR* minigene reporter assay following by hit compounds treatment. Splicing analysis is already explained in “Splicing reporter analysis” section.

### Statistics and reproducibility

Statistical tests, sample size, and number of biological replicates are reported in all the figure legends and/or described in the “Method” section. Statistical analyses were performed with GraphPad Prism 9.0 (GraphPad Software, Inc., San Diego, CA). The one-way analysis of variance (ANOVA) was used to compare between more than two groups. *p* values < 0.05 were considered to be statistically significant. Data were normally distributed with similar variance between the groups. All data described in graphical representations are mean ± standard error of the mean (SEM). The IC_50_ values were determined by analysis of dose-response inhibition using GraphPad prism.

### Reporting summary

Further information on research design is available in the Nature Portfolio Reporting Summary linked to this article.

## Supplementary information

Supplementary Information

Description of Additional Supplementary Files

Supplementary Data

Reporting summary

## Data Availability

Source data underlying the graphs presented in the main figures are available in the Supplementary Data files. Inquiry of any additional data should be requested from the corresponding author.
