# Supplementary material for: TDP-43 nuclear retention is antagonized by hypo-phosphorylation of its C-terminus in the cytoplasm
Source: Commun Biol. 2025 Jan 28;8:136. doi: 10.1038/s42003-025-07456-7 (PMC11775348; doi:10.1038/s42003-025-07456-7)
Supplement: Supplementary file 1 — Supplementary Information [file 42003_2025_7456_MOESM1_ESM.pdf]

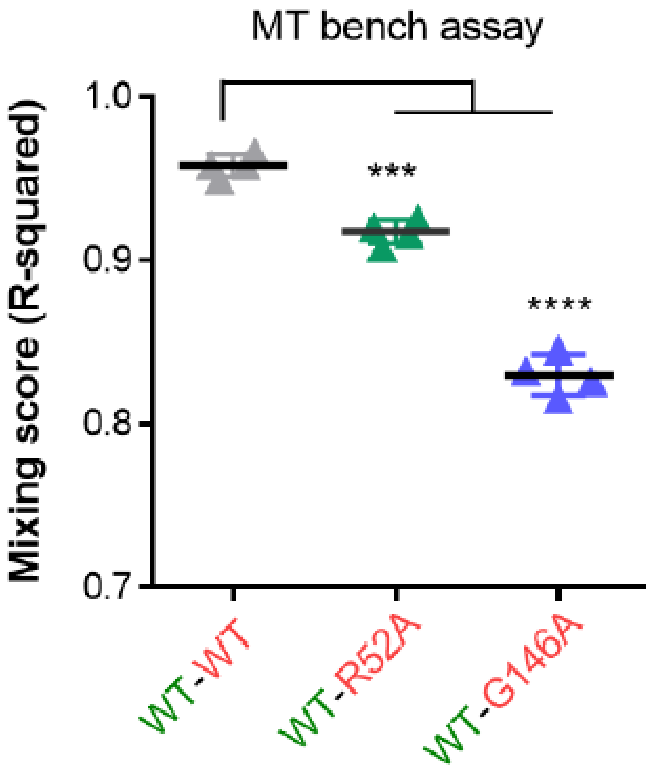

Supplementary Figure 1: Mixing score (R^2 values) of wild type TDP-43 and the indicated mutants measured along microtubules in HeLa cells using the microtubule in the MT bench assay (see Figure 1b). Error bars indicate SEM and the asterisks indicate statistical significance with ***p<0.001, ****p<0.0001, ns. non-significant, as measured by ANOVA test from n = 4 wells. R52A mutant induces an impaired multimerization of N-terminal domain of TDP-43. G146A mutant is no longer able to bind to GU repeats cooperatively such has WT TDP-43. Both mutations are thus expected to affect TDP-43 self-assemblies in the cell.

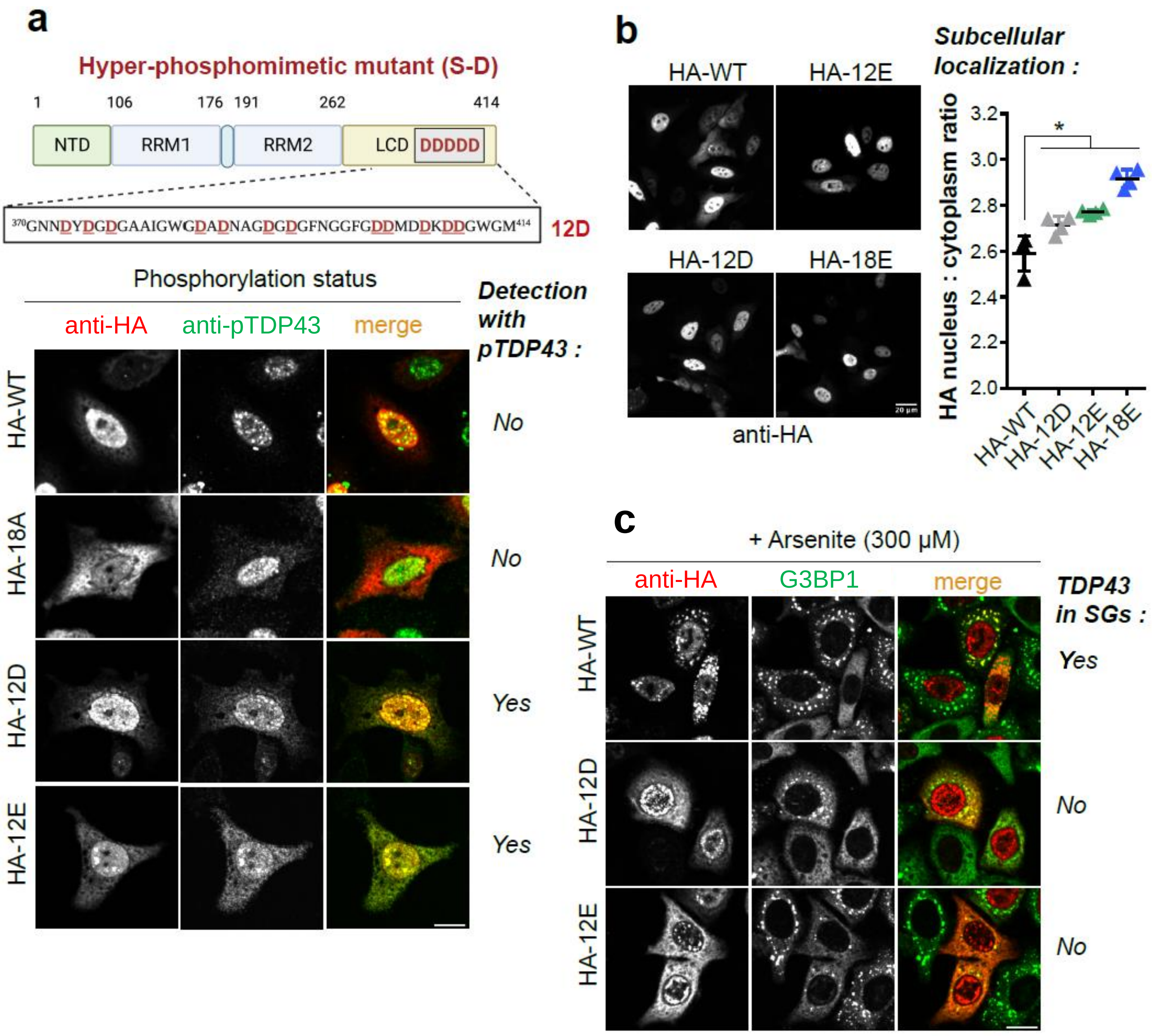

Supplementary Figure 2: Aspartate or glutamate hyper-phosphomimetic mutations display similar behaviors.

a. Presentation of TDP43 hyper-phosphomimetic mutant 12D. Lower panel: Images of HeLa cells expressing indicated HA-tagged protein and immune-stained with anti-phospho TDP43 antibody (Ser409/410). Hyper-phosphomimetic mutants are recognized by the antibody. Scale bar: 50 μm. **b.** Left panel: Subcellular distribution of indicated HA-tagged phosphomimetic mutants expressed in HeLa cells 24 h after transfection. Scale bar: 20 μm. Right panel: Measurement of the HA-tagged wild-type and hyper-phosphomimetic TDP-43 nuclear/cytoplasmic level. Each dot represents the mean value for cells in a single well (96-well plate). Error bars indicate SEM and the asterisks indicate statistical significance with **p<0.01,

***p<0.001, ****p<0.0001, as measured by ANOVA test from n = 4 wells for each mutant**. c.** Representative images of HeLa cells expressing HA-tagged phosphomimetic mutants (12D-HA or 12E-HA) and G3BP1-GFP after 1 h-exposure to 300 µM arsenite. G3BP1 in green; anti-HA in red. Scale bar: 50 µm.

a

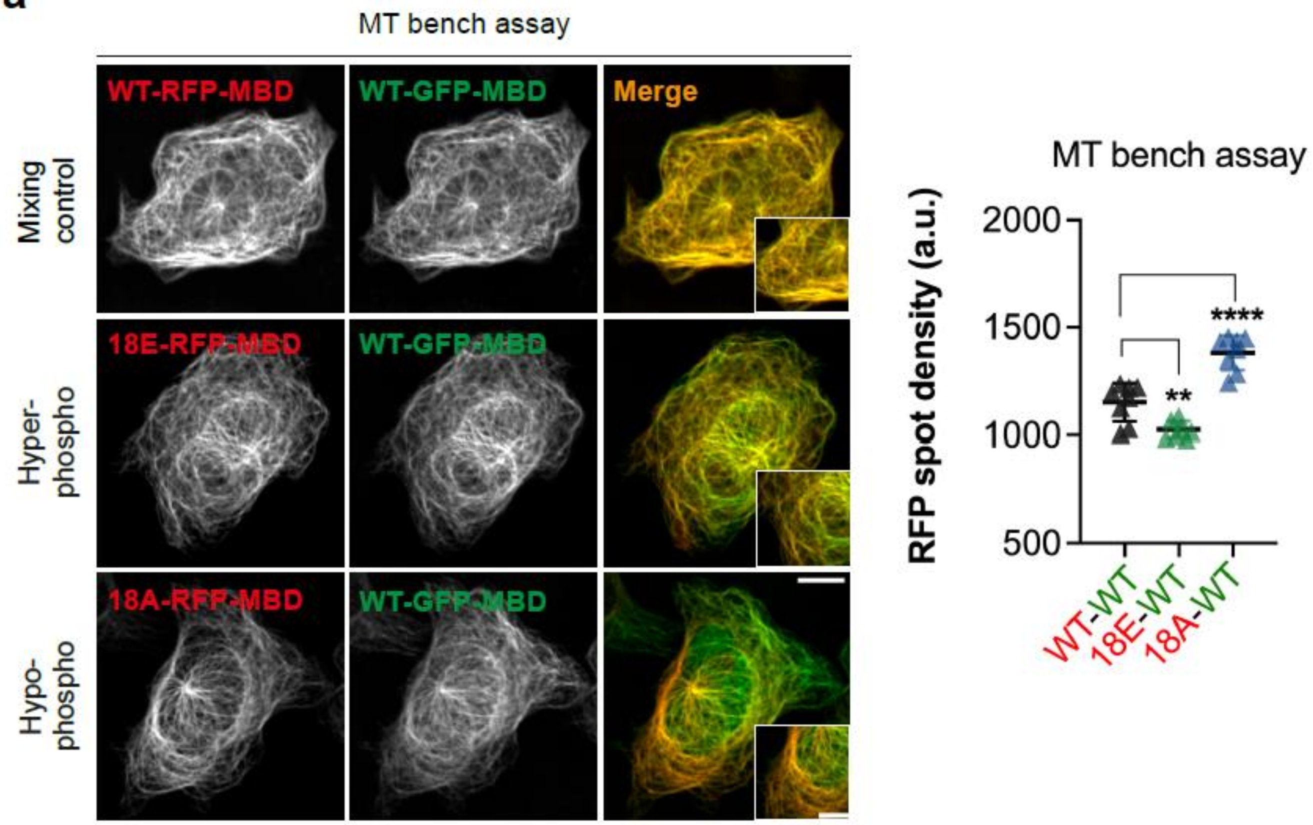

b

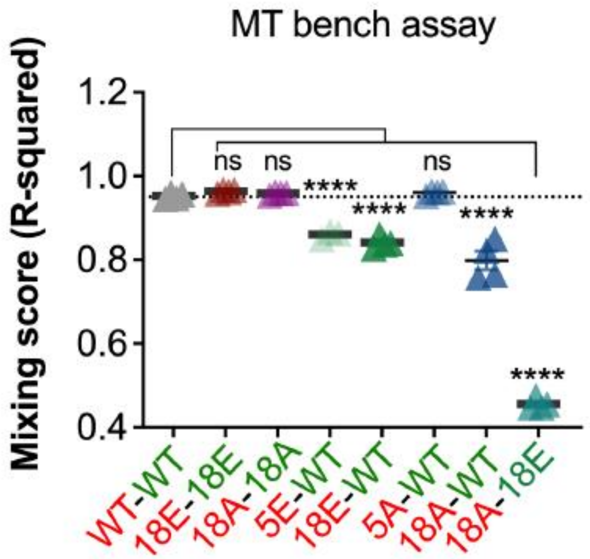

Supplementary Figure 3: Assessment of spot density from MT bench assay.
a. Left panel: Representative images of HeLa cells co-expressing indicated proteins used to measure their RFP spot density values. Scale bar: 30 µm. Zoom scale bar: 20 µm. Right panel: RFP spot density values (arbitrary unit) measured according to the indicated proteins. Note the

high RFP spot density values for 18A-RFP-MBD protein. Error bars indicate SEM and the asterisks indicate statistical significance with ***p*<0.01, ****p*<0.001, as measured by ANOVA test from *n* = 8 wells.

b. Mixing score (R^2 values) measured in HeLa cells co-expressing different TDP-43 RFP and GFP-tagged mutants, respectively as indicated (see Figure 1b). Error bars indicate SEM and the asterisks indicate statistical significance with ***p<0.001, ****p<0.0001, ns. non-significant, as measured by ANOVA test from n = 4 wells.

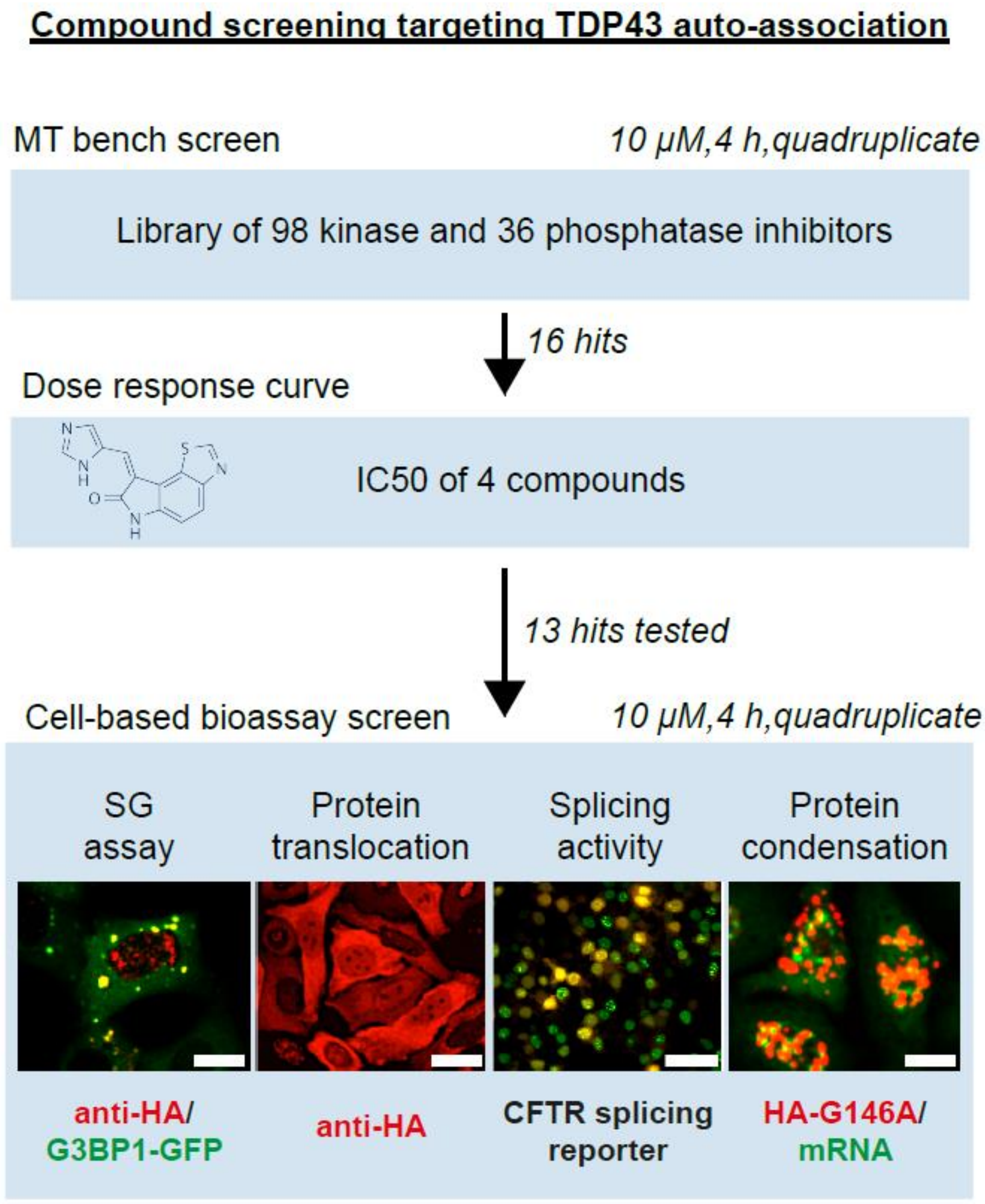

Supplementary figure 4: Screening strategy for identifying kinase/phosphatase inhibitors which significantly interfere with the phosphorylation-dependent self-assembly of TDP-43 C-terminus in the cytoplasm. 98 kinase and 36 phosphatase inhibitors were tested in a primary screen. Then, in secondary screens, the capacity of the selected compounds to alter the recruitment of TDP-43

in stress granules, its subcellular distribution, TDP-43-related mRNA splicing events and the cytoplasmic condensation of an mRNA-binding deficient TDP-43 mutant, G146A, was subsequently analyzed. Scale bar: 20 μm.

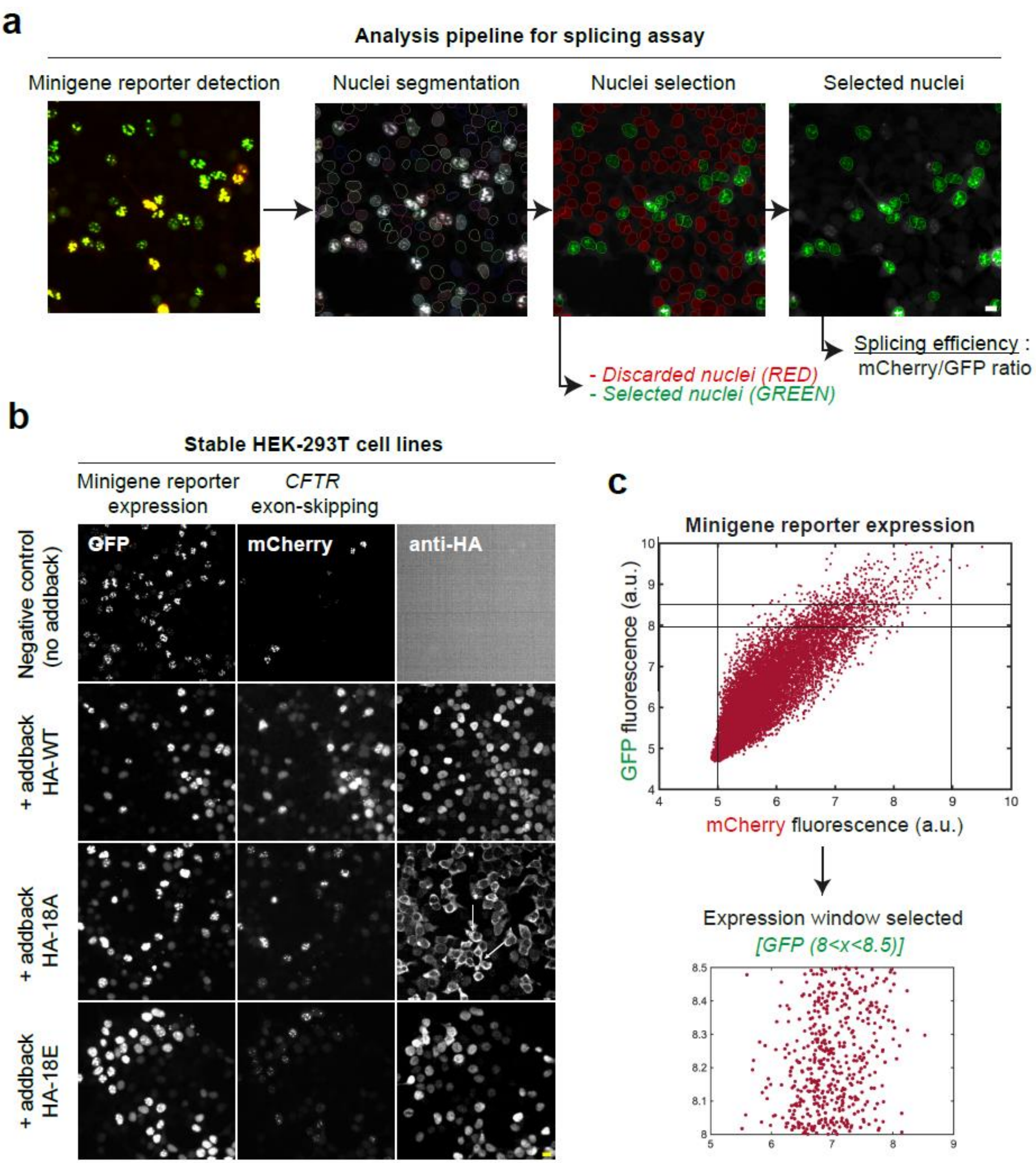

Supplementary Figure 5: Assessment of effect of phosphomimetic mutations in splicing assay.

a. Representative images of the splicing analysis method using the Harmony software associated to the HCS microscope. This pipeline allows the detection of HEK-293T cell nuclei that are filtered with average GFP and RFP intensity values higher than 2000 (a.u.) to calculate the mCherry:GFP ratio (splicing efficiency). Scale bar: 20 μm. **b.** Representative image of stable shRNA HEK-293T cells under indicated conditions. Note the cytoplasmic localization of 18A-HA (as indicated by arrow). Scale bar: 20 μm. **c.** Scatter plot of the relative GFP and RFP fluorescence intensity detected in all HEK-293T cells present in a single well under indicated conditions. A GFP expression window was selected for all conditions ($8<x<8.5$) to homogenize the data and eliminate the background.

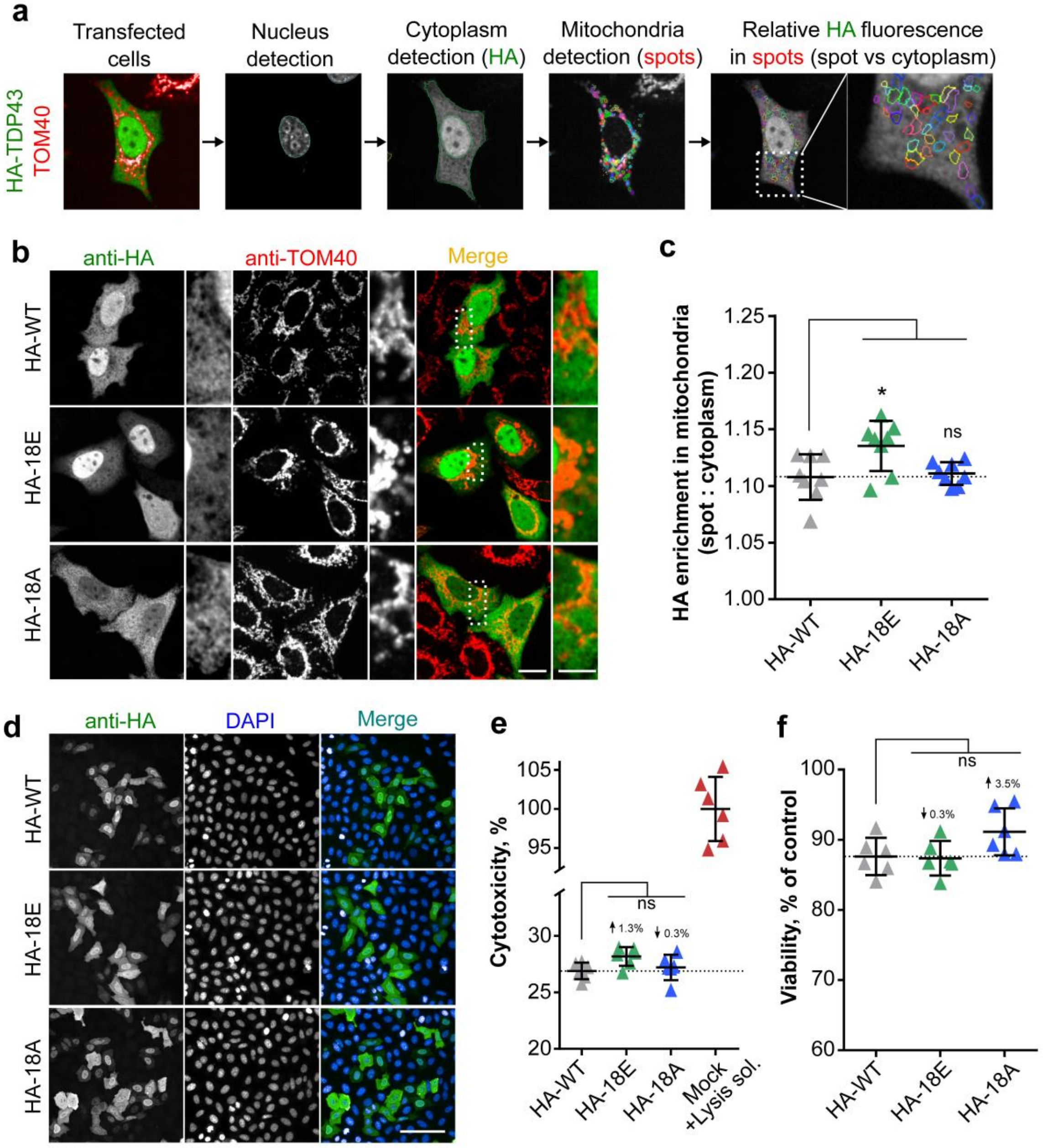

Supplementary Figure 6: Assessment of localization of phosphomimetic mutant in mitochondria.

a. Representative scheme of the automatic analysis of TDP-43 co-localization with mitochondria in the Harmony Software. This pipeline allows the detection of mitochondria using anti-TOM40 staining with the following measurement of the HA-tagged TDP-43 mutants in the corresponding mitochondria region. In HeLa cells transfected with the indicated plasmids, nuclei are first automatically detected, followed by cytoplasm in transfected cells, and finally spots that

correspond to TOM40-stained mitochondria. Finally, in the mitochondria region (spots), the fluorescent intensity of HA-TDP43 is measured and normalized to the cytoplasm intensity.
n (spots), the fluorescent intensity of HA-TDP43 is measured and normalized to the cytoplasm intensity.
b. Representative images of HeLa cells expressing HA-tagged wild-type or mutant TDP-43 used to quantify the relative protein enrichment in mitochondria defined via anti-TOM40 antibodies. Scale bar: 50 µm. Zoom scale bar: 20 µm.
c. Relative enrichment of HA-tagged wild-type and mutant TDP-43 in mitochondria.
Each dot represents the mean value over protein enrichment in mitochondria (mean per cell) in a single well (96-well plate). Error bars indicate SEM and the asterisks indicate statistical significance with $^*p<0.05$, ns. non-significant, as measured by ANOVA test from $n = 8$ wells for each mutant.
d. Representative images of HeLa cells expressing HA-tagged wild-type or mutant TDP-43 that were used to quantify the cytotoxicity measured by lactate dehydrogenase (LDH) released or viability estimated by MTT assay. Scale bar: 100 µm.
e. Percent cytotoxicity of HeLa cells transfected with HA-tagged wild-type or mutant TDP-43. Each dot represents the percent cytotoxicity per well (96-well plate). Cytotoxicity was measured using CytoTox-ONE™ homogenous membrane integrity assay (Promega) which assesses the level of released lactate dehydrogenase (LDH). The mean LDH release from mock-transfected cells treated with lysis solution was used as the maximum cytotoxic effect. Error bars indicate SD, ns. non-significant as measured by ANOVA test from $n = 6$ wells for each condition.
f. Percent viability of HeLa cells transfected with HA-tagged wild-type or mutant TDP-43. Each dot represents the percent viability per well (96-well plate). Viability was measured using the MTT assay allowing to probe the cellular metabolic activity. The mean of measurements from mock-transfected cells was used as the control. Error bars indicate SD, ns. non-significant as measured by ANOVA test from $n = 6$ wells for each condition.

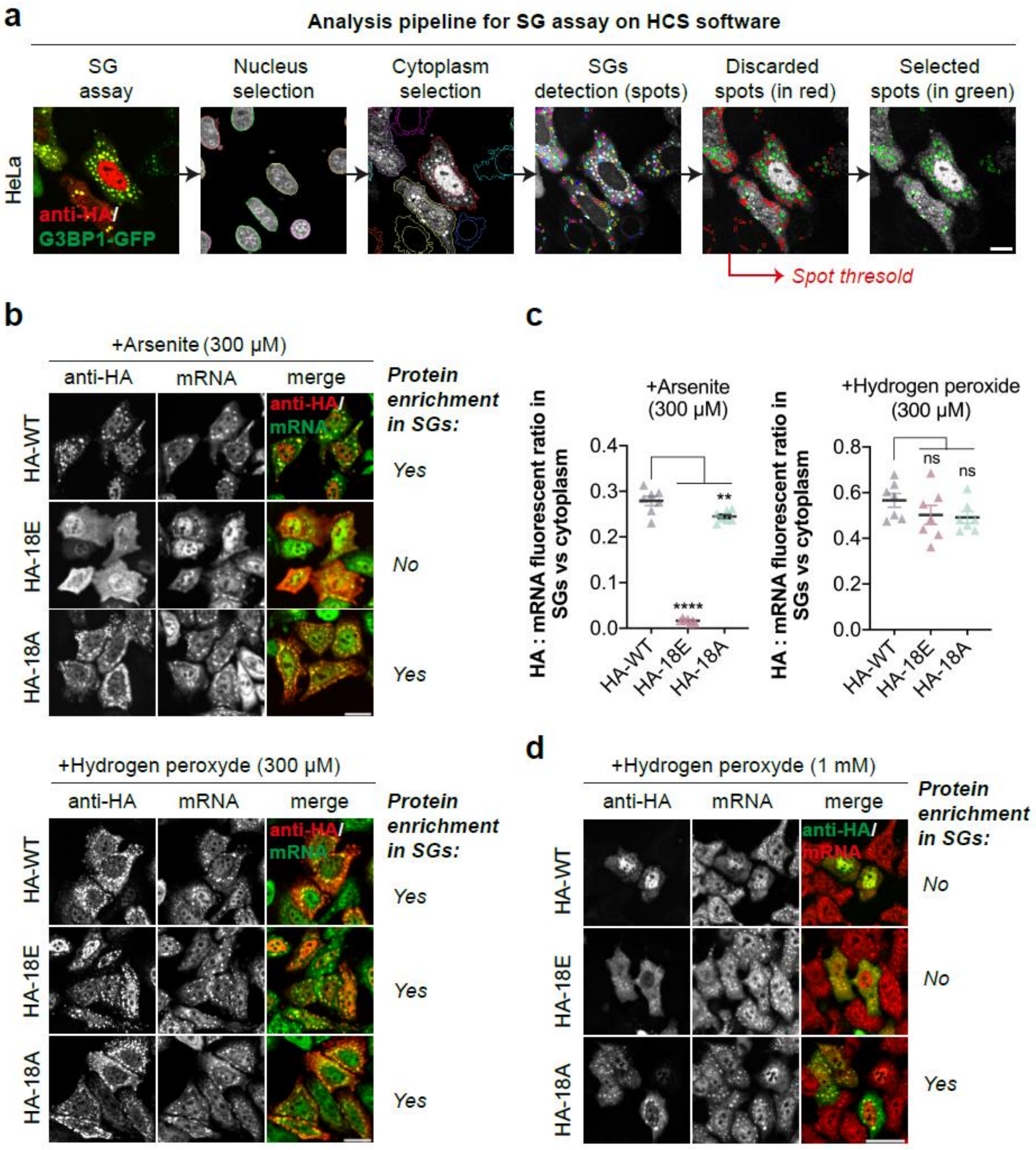

Supplementary Figure 7: Assessment of effect of phosphomimetic mutations in stress granules (SG) assay.

a. Representative images of the SG analysis method using the Harmony Software. This pipeline allows the detection of SGs (called spot). From HeLa cells co-transfected with the indicated plasmids, nuclei are first automatically detected, followed by cytoplasm, and finally spots. Spots

are filtered by area (<400 px^2), roundness (>0.8), length (<5 µm). The filtered spots are quantified. Scale bar: 20 µm. **b.** Representative images of HeLa cells expressing HA-tagged wild type or phosphomimetic mutants and mRNA used to quantify the relative protein enrichment of HA-tagged protein after 1 h-exposure to 300 µM arsenite or hydrogen peroxide. Note the loss of enrichment of 18E-HA after arsenite-type oxidative stress. Scale bar: 20 µm. **c.** Left panel: Automatic measurement of the SG/cytoplasm enrichment of HA-tagged TDP43 mutants divided by that of mRNA (in situ Hybridization). Each dot represents the mean value over stress granules detected in a single well (96-well plate). Error bars indicate SEM and the asterisks indicate statistical significance with $^{**}p<0.01$, $^{****}p<0.0001$, ns. non-significant, as measured by ANOVA test from $n = 7$ wells. Right panel: Quantification of the recruitment of HA-tagged wild-type TDP43 or phosphomimetic mutants in SGs after 1 h-exposure to 300 µM hydrogen peroxide. Note the absence of effect of hydrogen peroxide-type oxidative stress on the recruitment of HA-tagged protein in SGs. ns. non-significant, ANOVA test from n= 7 wells. **d.** Representative images of HeLa cells expressing HA-tagged wild-type TDP43 or phosphomimetic mutants and mRNA to quantify the relative protein enrichment of HA-tagged protein after 1h-exposure to 300 µM (upper panel) and 1 mM (center panel) hydrogen peroxide (H_2O_2). Note the enrichment of 18A-HA in the SGs despite the high hydrogen peroxide concentrations. However, high concentrations of H_2O_2 (>300 µM) prevent the recruitment of wild type and TDP-43 hyper-phosphomimetic mutant in stress granules in the cytoplasm. Scale bar: 50 µm.

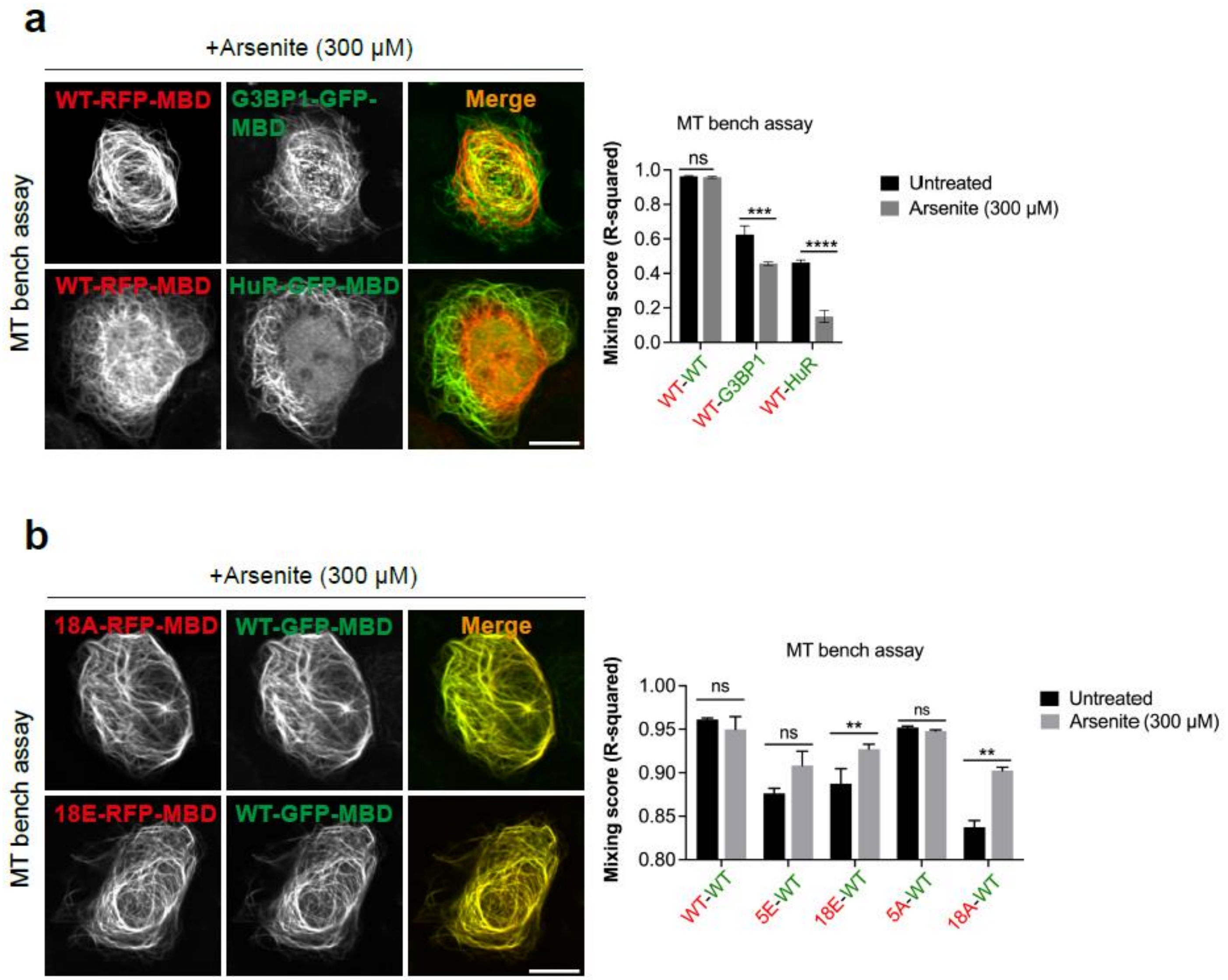

Supplementary Figure 8: MT bench assay to probe the TDP-43 aggregation process. **a.** Left panel: Representative images of HeLa cell co-expressing indicated proteins after 1 h-exposure to 300 µM arsenite to measure their mixing along the microtubule network (MT bench assay). Scale bar: 50 µm. Right panel: Mixing score (R^2 values) measured in HeLa cells expressing wild type TDP-43 and the indicated wild-type RNA-binding proteins (G3BP1 and HuR). Note the important de-mixing of these RBPs with the wild-type TDP43. Error bars indicate SEM and the asterisks indicate statistical significance with ***p<0.001, ****p<0.0001, ns. non-significant, as measured by Student's t-test from $n = 4$ wells. **b.** Left panel: Representative images of HeLa cell co-expressing indicated proteins after 1 h-exposure to 300 µM arsenite to measure their mixing along the microtubule network (MT bench assay). Scale bar: 50 µm. Right panel: Mixing score (R^2 values) measured in HeLa cells expressing wild type TDP-43 and the indicated phosphomimetic TDP-43 mutants. Note the opposite effect with phosphomimetic mutants. Error bars indicate SEM and the asterisks indicate statistical significance with *p<0.01, *p<0.01, ns. non-significant, as measured by Student's t-test from $n = 4$ wells.

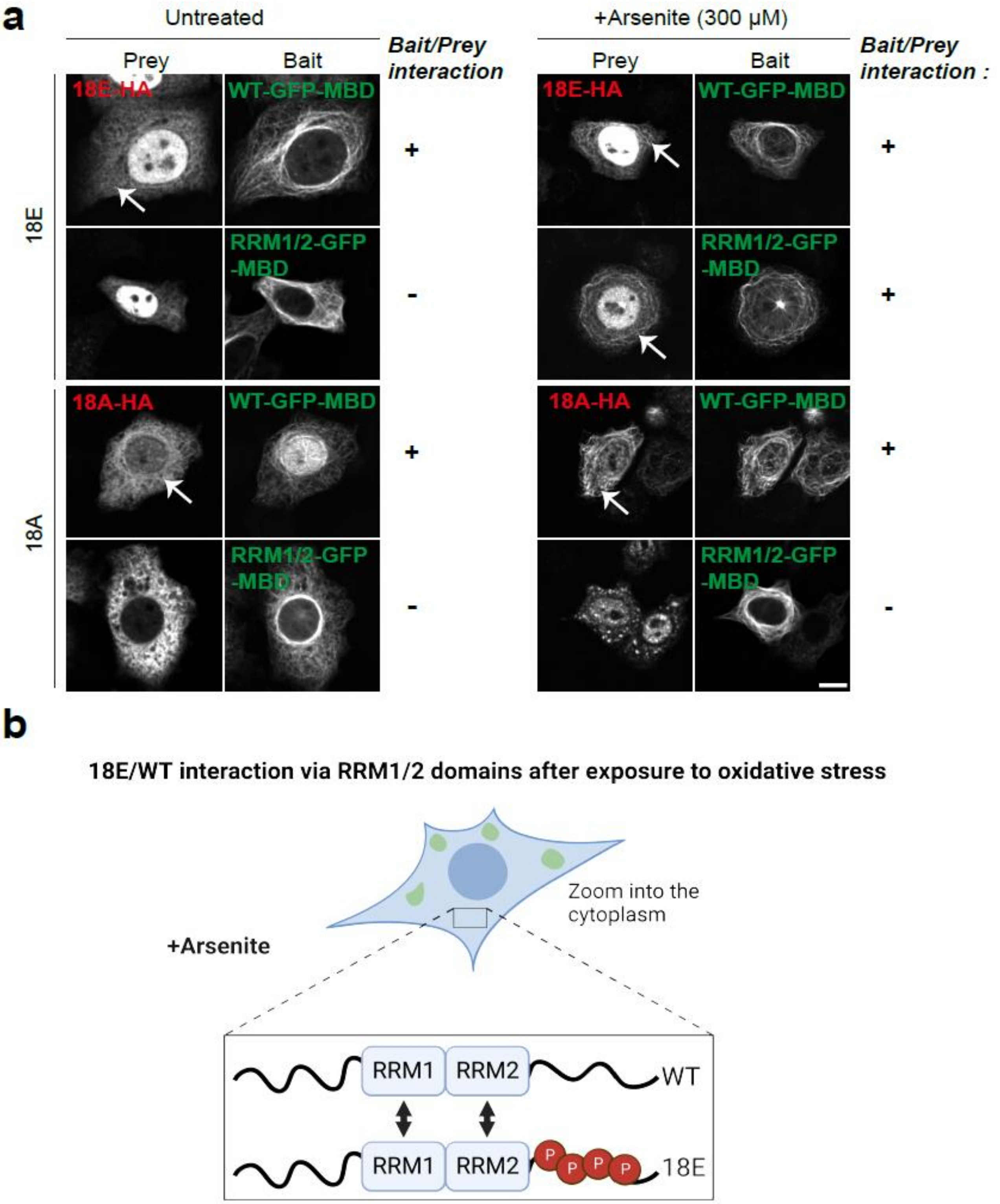

Supplementary Figure 9: TDP-43 RRM1/2 domains was brought as a bait onto microtubules to probe the recruitment onto microtubules of hyper- and hypo-phosphomimetic mutants, used as the prey, in the presence of arsenite.

a. Representative images of HeLa cells co-expressing HA-tagged phosphomimetic mutants as prey and the indicated TDP43 mutant fused to microtubule-binding domain as bait to probe the

interaction between bait/prey in the presence or absence of arsenite (300 μM, 1 h). Note the hyper-phosphomimetic (18E) mutant is recruited onto microtubules in the presence of arsenite but not the hypo-phosphomimetic (18A) mutant. Scale bar: 20 μm. **b.** Schematic view representing the results obtained with the Bait/Prey interaction assay. Under acute oxidative stress, including arsenite stress, the hyper-phosphomimetic mutant (18E) interact with wild type TDP-43 through their RRM domains leading to the exclusion of TDP-43 from stress granules and found in the cytoplasm as 18E mutant. However, in the same stress conditions, the hypo-phosphomimetic mutant is found located in the stress granules in the cytoplasm. This panel was Created in BioRender. Rattenbach, R. (2025) https://BioRender.com/f43j392.

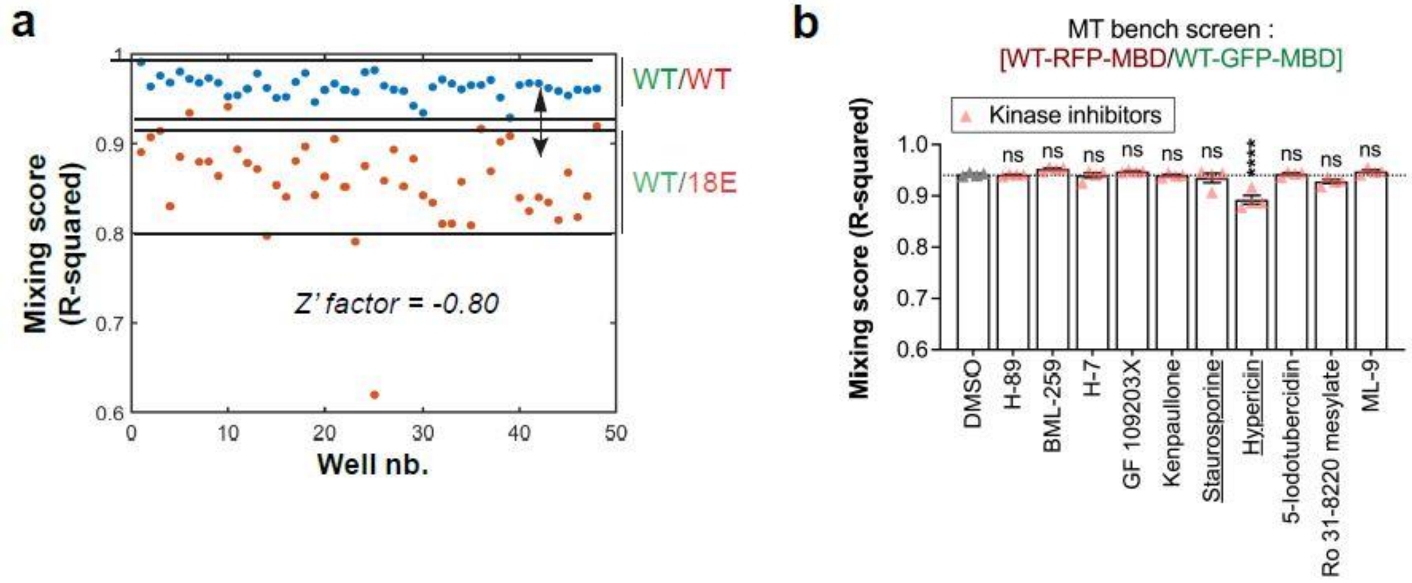

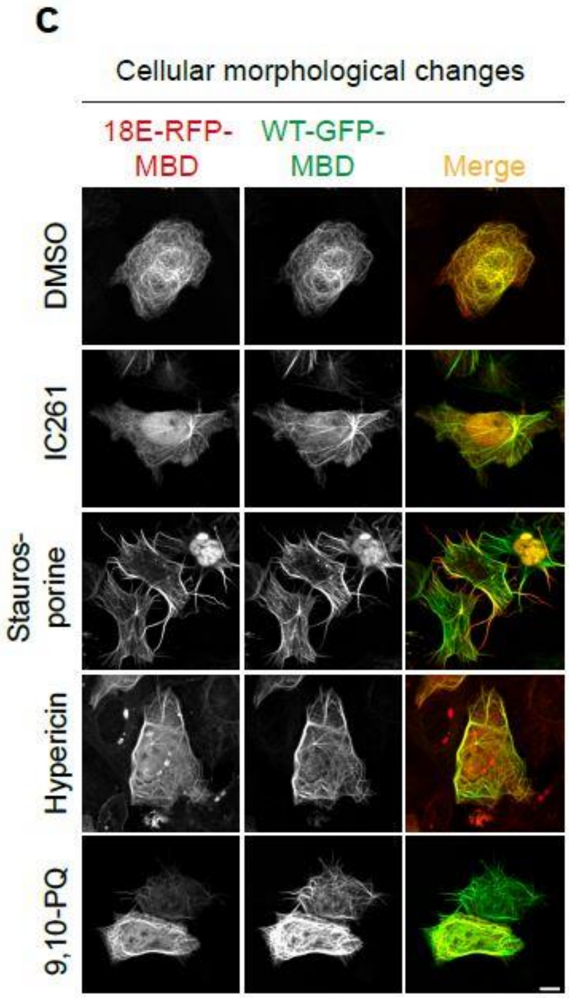

d

Molecule	CAS number	Inhibitor type	Target name	Mixing score (R^2) at 10µM	Measured IC50 [nM]	Activity at 1µM (Yes/No)	Toxicity in HeLa cells (Yes/No)
Hypericin	548-04-9	Kinase	PKC	0.54		Yes	Yes
Staurosporine	62996-74-1	Kinase	PKC	0.59		Yes	Yes
*Ro 31-8220 mesylate	138489-18-6	Kinase	PKC	0.73	1279	Yes	No
*5-Iodotubercidin	24386-93-4	Kinase	Adenosine kinase	0.74	533	Yes	No
GF109203X	133052-90-1	Kinase	PKC	0.75		No	No
Kenpaullone	142273-20-9	Kinase	GSK-3beta	0.78		No	No
H-89 2HCl	127243-85-0	Kinase	PKA	0.80		No	No
H-7 2HCl	108930-17-2	Kinase	PKA	0.81		No	No
BML-259	267654-00-2	Kinase	Cdk5	0.82		No	No
ML-9	105637-50-1	Kinase	MLCK	0.81		No	No
*C16 PKRi	608512-97-6	Kinase	PKR	0.74	1211	Yes	No
IC261	186611-52-9	Kinase	CK1	0.79		No	Yes
*JNJ-7706621	443797-96-4	Kinase	Aurora A/B CDK1/2	0.76	2179	No	No
Linifanib	796967-16-3	Kinase	VEGFR	0.82		No	No
Alexidine 2HCL	1715-30-6	Phosphatase	PTPMT1	0.88		No	No
9,10-PQ	84-11-7	Phosphatase	PTPN1	0.88		No	Yes

Supplementary Figure 10: Assessment of effect of kinase inhibitors on mixing score of wild type TDP-43 with the hyper-phosphomimetic mutant (18E).

a. Mixing score of wild-type TDP43 in a 96-well plate. Each condition was run in 48 wells. *Z'* factor is -0.80, which corresponds to a very bad assay for compounds increasing the mixing score. There is too much overlap between the WT/WT and WT/18E for the assay to be useful. Note that this is the reason why we did not choose these conditions for the MT bench screen. **b.** Mixing score (R^2 values) of WT TDP-43 with itself measured with the MT bench assay in HeLa cells. Note that no compound can change the mixing of TDP-43 with itself, as expected, except hypericin due to morphological changes or toxicity. Error bars indicate SEM and the asterisks indicates statistical significance with ****$p<0.0001$, ns. non-significant, as measured by ANOVA test from $n = 4$ wells. **c.** Representative images of HeLa cells co-expressing the indicated proteins after treatment with indicated hit compounds. Scale bar: 30 μm. **d.** Summary table of hit compounds discovered using the MT bench assay over the screening system.

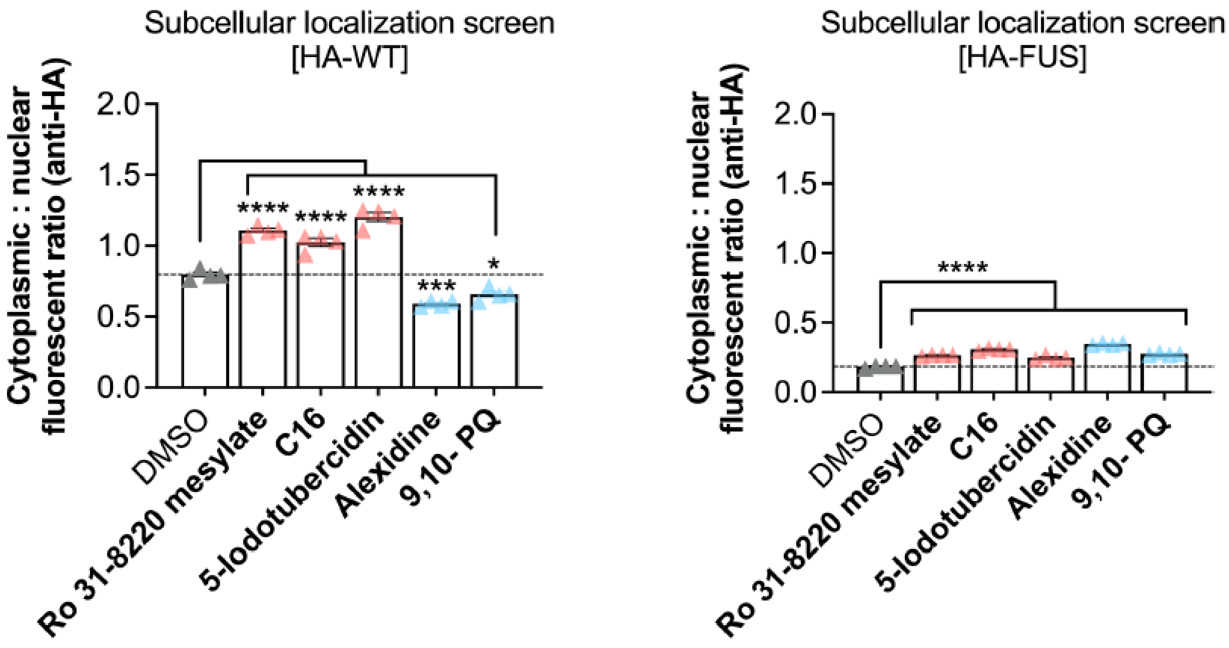

Supplementary Figure 11: Differential effect of selected kinase/phosphatase inhibitors on subcellular localization of TDP-43 and FUS proteins. Left panel: Automatic measurement of the cytoplasmic:nuclear ratio of HA-tagged TDP-43 under indicated conditions. Each data point represents the mean ratio value obtained from a single well in a 96-well plate (see Figure 6a, Middle panel). Right panel: Automatic measurement of the cytoplasmic:nuclear ratio of HA-tagged FUS under indicated conditions. Each data point represents the mean ratio value obtained from a single well in a 96-well plate. Error bars indicate SEM and the asterisks indicate statistical significance with *$p<0.05$, **$p<0.01$, ***$p<0.001$, ****$p<0.0001$, ns. non-significant as measured by ANOVA test from $n = 4$ wells.

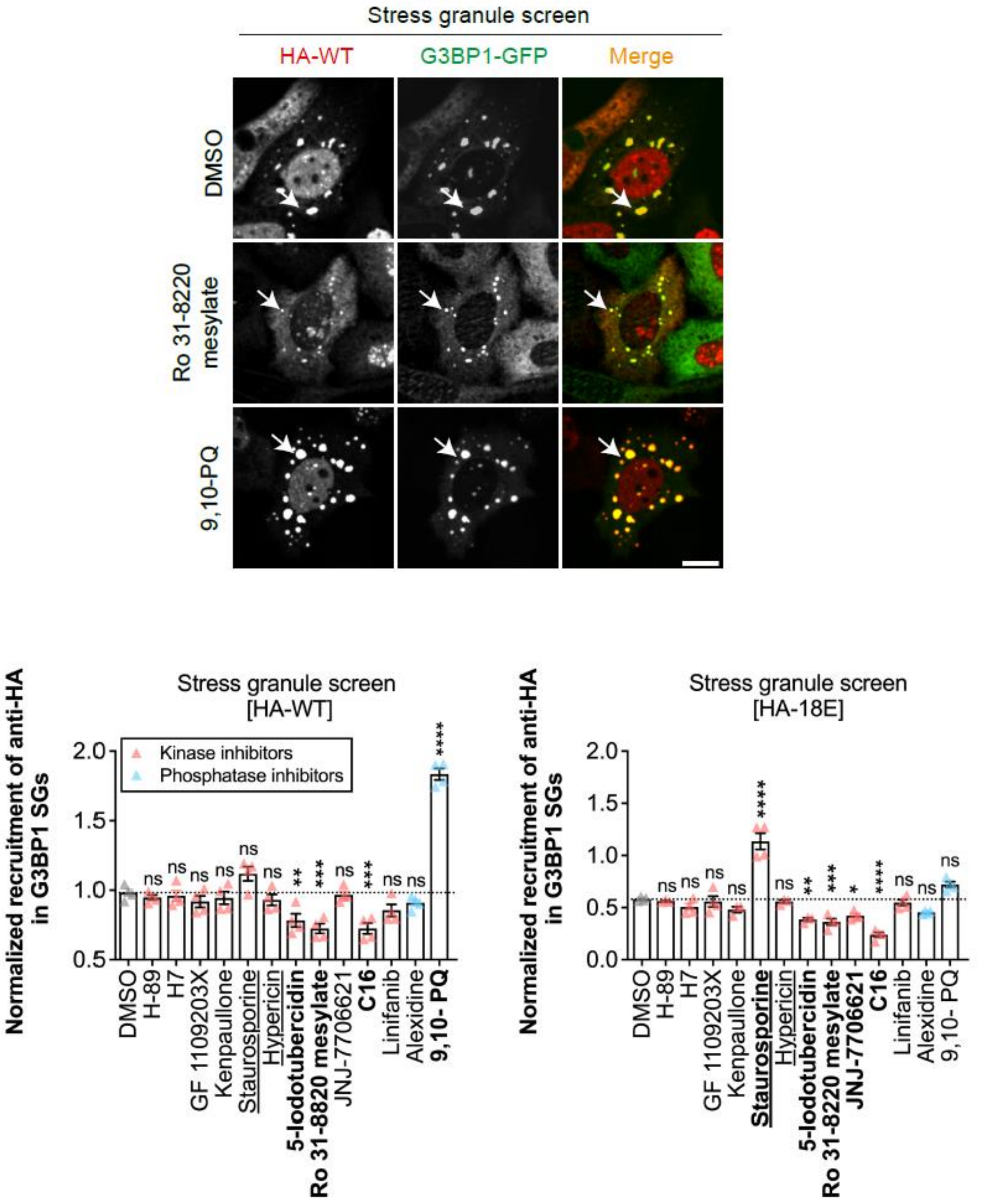

Supplementary Figure 12: Effect of selected compounds on recruitment of HA-tagged wild type TDP-43 in stress granules. Upper panel: Recruitment of HA-tagged wild type TDP-43 in stress granules in HeLa cells expressing GFP-G3BP1 and treated with the indicated compounds during 4 h (10 µM). Anti-HA in red. Arrows indicate representative stress granules. Scale bar: 20

μm. Lower panels: Automatic measurement of the relative enrichment of HA-antibody/GFP fluorescence in stress granules versus cytoplasm under indicated conditions. Each data point represents the mean ratio value obtained from a single well in a 96-well plate. As control, the same ratio was measured with the hyper-phosphomimetic TDP-43 mutant (18E) under the same condition. Error bars indicate SEM and the asterisks indicate statistical significance with $*p<0.05$, $**p<0.01$, $***p<0.001$, $****p<0.0001$, ns. non-significant as measured by ANOVA test from $n = 4$ wells.

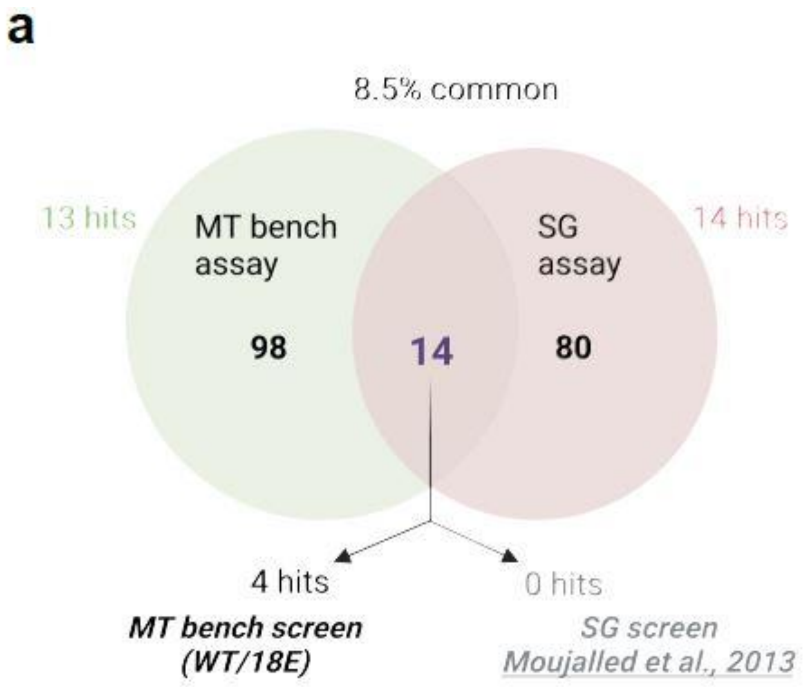

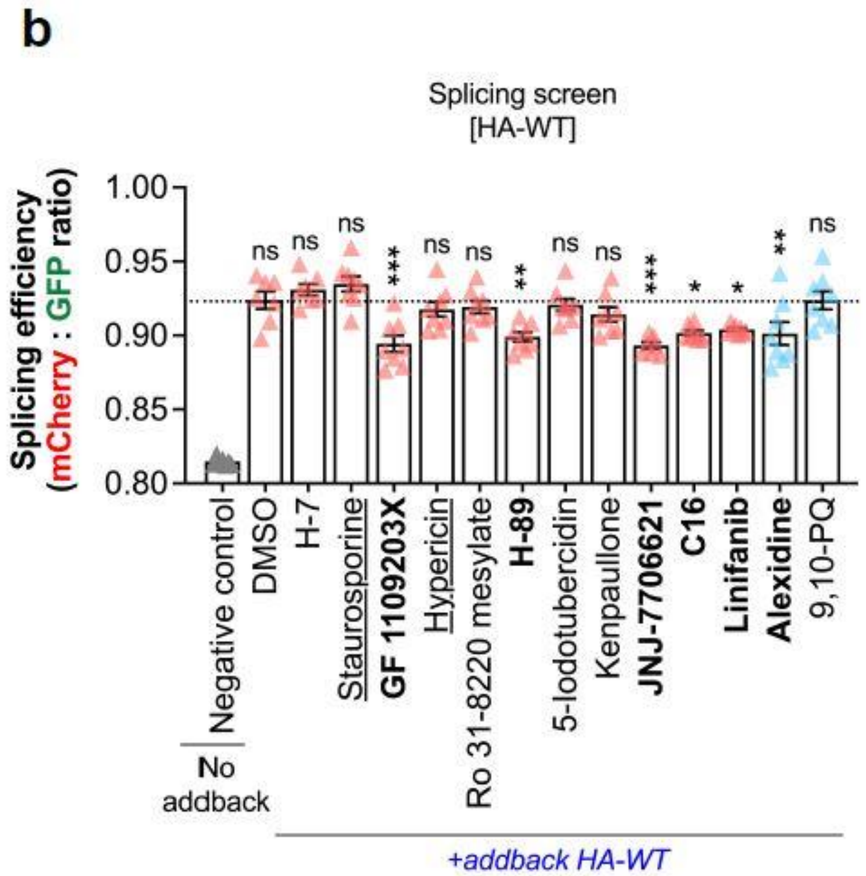

197 **Supplementary Figure 13: Assessment of effect of the compound hits on splicing TDP-43-dependent**
198 **assay.**

a. Venn diagram showing the total number of kinase inhibitors tested for MT bench assay and SGs assay (98 and 80, respectively), together with those commonly tested in both screens (14 which represents 8.5 % total). From these 14 common kinase inhibitors, 4 hits were obtained from the MT bench assay and none from the SGs assay. Independently of these common 14 molecules, hits were obtained from both assays (13 and 14 hits from MT bench and SGs, respectively). **b.** Quantitative analysis of the splicing efficiency under indicated condition at the single cell level and after treatment with the indicated compounds during 4 h (10 µM). Each dot represents the average values in selected HEK-293T cells in a single well. Error bars indicate SEM and the asterisks indicates statistical significance with *$p<0.05$, **$p<0.01$, ***$p<0.001$, ns. non-significant, as measured by ANOVA from $n = 8$ wells.

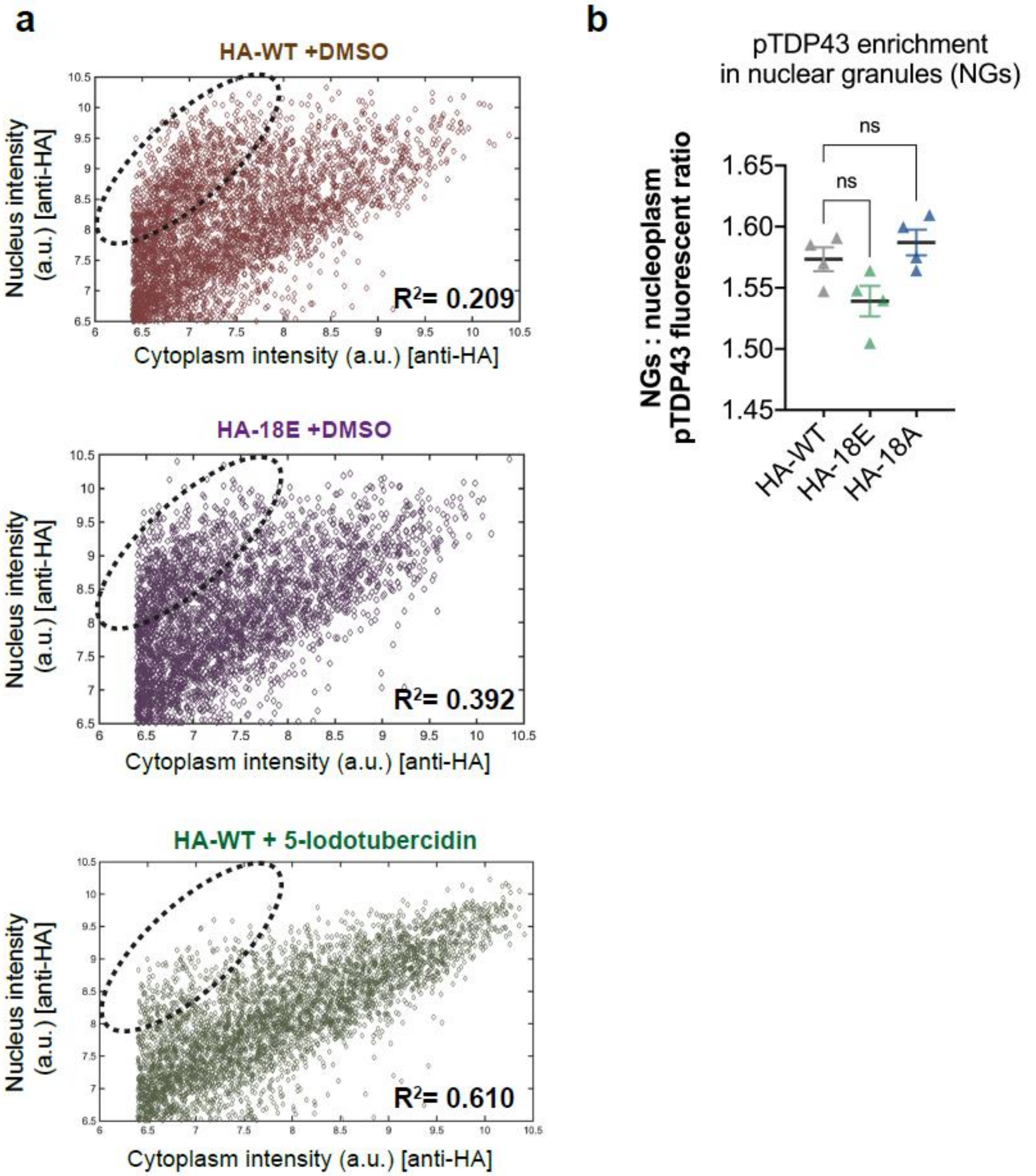

Supplementary Figure 14: Subcellular distribution of TDP-43 and phosphomimetic mutants.

a. Scatter plot of subcellular distribution of indicated HA-tagged TDP43 treated with DMSO or kinase inhibitor. A linear regression shows that in HeLa cells expressing wild-type TDP43 treated with 5-Iodotubercidin kinase inhibitors, a fraction of the cell with increased TDP43 cytoplasmic level with a significant decrease in the nuclear fraction (see circle). Each dot

represents a single cell. R^2 are indicated for each condition. **b.** Quantification of the nuclear granule:nucleoplasm intensity ratio from cells expressing the indicated HA tagged TDP43 and stained with anti-phospho TDP-43 antibody (Ser 409/410). ns. non-significant, as measured by ANOVA from $n = 4$ wells.

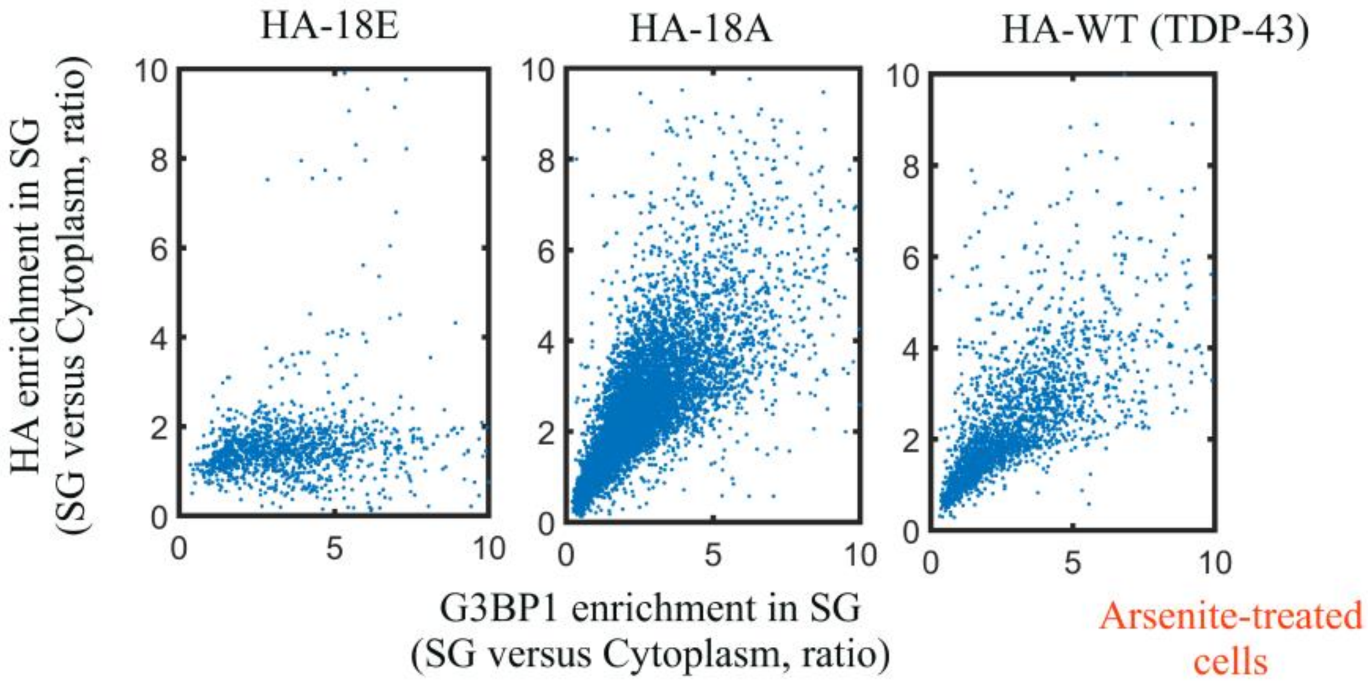

Supplementary Figure 15: Relative enrichment of HA-tagged TDP-43 in G3BP-GFP-rich granules.
Without additional stress and in HeLa cells expressing both indicated HA-tagged TDP-43 mutants and GFP-labeled stress granules, we measured both the enrichment of GFP (x axis) and anti-HA intensity (y axis) in stress granules versus cytoplasm. Each dot represents the value obtained for one SG detected in cells in a representative well. When the slope value is larger than zero, it means that HA-tagged TDP-43 is significantly recruited in stress granules. The recruitment is clearly observable for WT-TDP-43 and 18A but not for 18E. Using this plot, we can measure the slope for a single well as displayed in figure 4a.

Supplementary Tables:

Table S1. Plasmids used for MT bench assay

Name	Sequence	Tag	References
TDP-43	FL (1-414)	RFP-MBD or<br>GFP-MBD	Maucuer, et *al*. J Cell Sci. 2018
TDP-43	FL (1-414)	GFP	Boca et al., 2015
G3BP1	FL (1-466)	GFP-MBD	Maucuer, et *al*. J Cell Sci. 2018
G3BP1	FL (1-466)	GFP	Boca et al., 2015
HuR	FL (1-326)	GFP-MBD	Maucuer, et *al*. J Cell Sci. 2018
RRM1/2 (TDP-43 RRM1/2)	98-264	GFP-MBD	Rengifo et al. (2021)

Table S2. Primers used to construct TDP-43 hyper- or hypo-phosphorylated mutants for MT bench experiments:

Name	Tag	Sense primer (bearing NdeI restriction site)	Antisense primer<br>(bearing AscI restriction site)
5E	RFP-MBD	GTAATGTCACAGCGA CATATGATAG	GCGGGCGCGCCAACA TTCCCCAGCCAGAAG ACTTAGAATCC
18E	RFP-MBD	GTAATGTCACAGCGA CATATGATAG	ATTGCGGGCGCGCCA ACATTCC
18E	GFP-MBD	GTAATGTCACAGCGA CATATGATAG	ATTGCGGGCGCGCCA ACATTCC
5A	RFP-MBD	GTAATGTCACAGCGA CATATGATAG	GCGGGCGCGCCAACA TTCCCCAGCCAGAAG ACTTAGAATCC
18A	RFP-MBD	GTAATGTCACAGCGA CATATGATAG	ATTGCGGGCGCGCCA ACATTCC
12D	RFP-MBD	GTAATGTCACAGCGA CATATGATAG	ATT GCG GGC GCG CCA ACA TTC C
12E	RFP-MBD	GTAATGTCACAGCGA	ATTGCGGGCGCGCCA

		CATATGATAG	ACATTCC

Table S3. Alignement of C-terminal part of wild type TDP-43 and its hyper- or hypo- phosphomimetic mutants.

Alignement of C-terminal part of wild type TDP-43 and its hyper-phosphomimetic mutants with substitution of serine by glutamic residue (showed in red):

```
TDP-43(WT)  KQSQDEPLRSRKVFVGRCTEDMTEDELREFFSQYGDVMDVFIPKPFRAFAFVTFADDQIA 240
5E          KQSQDEPLRSRKVFVGRCTEDMTEDELREFFSQYGDVMDVFIPKPFRAFAFVTFADDQIA
18E         KQSQDEPLRSRKVFVGRCTEDMTEDELREFFSQYGDVMDVFIPKPFRAFAFVTFADDQIA


TDP-43(WT)  QSLCGEDLIIKGISVHISNAEPKHNSNRQLERSGRFGGNPGGFGNQGGFGNSRGGGAGLG 300
5E          QSLCGEDLIIKGISVHISNAEPKHNENRQLEREGRFGGNPGGFGNQGGFGNERGGGAGLG
18E         QSLCGEDLIIKGISVHISNAEPKHNSNRQLERSGRFGGNPGGFGNQGGFGNSRGGGAGLG


TDP-43(WT)  NNQGSNMGGGMNFGAFSINPAMMAAAQAALQSSWGMMGMLASQQNQSGPSGNNQNQGNMQ 360
5E          NNQGENMGGGMNFGAFEINPAMMAAAQAALQSSWGMMGMLASQQNQSGPSGNNQNQGNMQ
18E         NNQGSNMGGGMNFGAFSINPAMMAAAQAALQEEWGMMGMLAEQQNQEGPEGNNQNQGNMQ

TDP-43(WT)  REPNQAFGSGNNSYSGSNSGAAIGWGSASNAGSGSGFNGGFGSSMDSKSSGWGM 414
5E          REPNQAFGSGNNSYSGSNSGAAIGWGSASNAGSGSGFNGGFGSSMDSKSSGWGM
18E         REPNQAFGEGNNSYEGENEGAAIGWGEAENAGEGEGFNGGFGEEMDEKEEGWGM
```

Alignement of C-terminal part of wild type TDP-43 and its hypo-phosphomimetic mutants with substitution of serine by alanine residue (showed in blue):

```
TDP-43      KQSQDEPLRSRKVFVGRCTEDMTEDELREFFSQYGDVMDVFIPKPFRAFAFVTFADDQIA 240
5A          KQSQDEPLRSRKVFVGRCTEDMTEDELREFFSQYGDVMDVFIPKPFRAFAFVTFADDQIA
18A         KQSQDEPLRSRKVFVGRCTEDMTEDELREFFSQYGDVMDVFIPKPFRAFAFVTFADDQIA

TDP-43      QSLCGEDLIIKGISVHISNAEPKHNSNRQLERSGRFGGNPGGFGNQGGFGNSRGGGAGLG 300
5A          QSLCGEDLIIKGISVHISNAEPKHNANRQLERAGRFGGNPGGFGNQGGFGNARGGGAGLG
18A         QSLCGEDLIIKGISVHISNAEPKHNSNRQLERSGRFGGNPGGFGNQGGFGNSRGGGAGLG

TDP-43      NNQGSNMGGGMNFGAFSINPAMMAAAQAALQSSWGMMGMLASQQNQSGPSGNNQNQGNMQ 360
5A          NNQGANMGGGMNFGAFAINPAMMAAAQAALQSSWGMMGMLASQQNQSGPSGNNQNQGNMQ
18A         NNQGSNMGGGMNFGAFSINPAMMAAAQAALQAAWGMMGMLAAQQNQAGPAGNNQNQGNMQ

TDP-43      REPNQAFGSGNNSYSGSNSGAAIGWGSASNAGSGSGFNGGFGSSMDSKSSGWGM 414
5A          REPNQAFGSGNNSYSGSNSGAAIGWGSASNAGSGSGFNGGFGSSMDSKSSGWGM
18A         REPNQAFGAGNNSYAGANAGAAIGWGAAANAGAGAGFNGGFGAAMDAKAAGWGM
```

Alignement of C-terminal part of wild type TDP-43 and its hyper-phosphomimetic mutants with substitution of serine by aspartic or glutamic acid residues (red):

```
TDP-43(WT) KQSQDEPLRSRKVFVGRCTEDMTEDELREFFSQYGDVMDVFIPKPFRAFAFVTFADDQIA 240
12D        KQSQDEPLRSRKVFVGRCTEDMTEDELREFFSQYGDVMDVFIPKPFRAFAFVTFADDQIA
12E        KQSQDEPLRSRKVFVGRCTEDMTEDELREFFSQYGDVMDVFIPKPFRAFAFVTFADDQIA

TDP-43(WT) QSLCGEDLIIKGISVHISNAEPKHNSNRQLERSGRFGGNPGGFGNQGGFGNSRGGGAGLG 300
12D        QSLCGEDLIIKGISVHISNAEPKHNSNRQLERSGRFGGNPGGFGNQGGFGNSRGGGAGLG
12E        QSLCGEDLIIKGISVHISNAEPKHNSNRQLERSGRFGGNPGGFGNQGGFGNSRGGGAGLG

TDP-43(WT) NNQGSNMGGGMNFGAFSINPAMMAAAQAALQSSWGMMGMLASQQNQSGPSGNNQNQGNMQ 360
12D        NNQGSNMGGGMNFGAFSINPAMMAAAQAALQSSWGMMGMLASQQNQSGPSGNNQNQGNMQ
12E        NNQGSNMGGGMNFGAFSINPAMMAAAQAALQSSWGMMGMLASQQNQSGPSGNNQNQGNMQ

TDP-43(WT) REPNQAFGSGNNSYSGSNSGAAIGWGSASNAGSGSGFNGGFGSSMDSKSSGWGM 414
12D        REPNQAFGSGNNSYDGDNDGAAIGWGDADNAGDGDGFNGGFGDDMDDKDDGWGM
12E        REPNQAFGSGNNSYEGENEGAAIGWGEAENAGEGEGFNGGFGEEMDEKEEGWGM
```

Table S4. Primers used for to construct TDP-43 mutants in HA-tag plasmid.

**Plasmid name**	**Tag**	**Sense Primer (bearing NdeI restriction site)**	**Antisense primer (bearing XhoI restriction site)**
HA-5E	HA	GTAATGTCACAGCGACATATGATAG	AATTTCTCGAGTTACATTCCCCAGCCAGAAGACTTAGAATCC
HA-18E	HA	GTAATGTCACAGCGACATATGATAG	AATTTCTCGAGTTACATTCCCCAGCCTTCCTCCTTTTCATCC
HA-5A	HA	GTAATGTCACAGCGACATATGATAG	AATTTCTCGAGTTACATTCCCCAGCCAGAAGACTTAGAATCC
HA-18A	HA	GTAATGTCACAGCGACATATGATAG	AATTTTCTCGAGTTACATTCCCCAGCCAGCTGCCTTAGCATCC
HA-12D	HA	GTAATGTCACAGCGACATATGATAG	GAT ATA GTC GAG TTA CAT TCC CCA GCC ATC ATC CTT ATC ATC
HA-12E	HA	GTAATGTCACAGCGACATATGATAG	AATTTCTCGAGTTACATTCCCCAGCCTTCCTCCTTTTCATCC

Table S5. Sequence of shRNA from the TRC library used in this study:

Gene name	shRNA name	Type	Sequence
TARDBP	TRCN0000016038	TRC human shRNA (Lentiviral)	GCTCTAATTCTGGTGCAGCAA

Table S6. Primers used for shRNA resistance (splicing assay)

Plasmid name	Tag	Sense primer	Anti-sense primer
HA-rTDP-43 (WT)	HA	GTGGCTCTAATTCTGGGGCGGC GATTGGTTGGGGATCAGC	GCTGATCCCCAACCAATCGCCGC CCCAGAATTAGAGCCAC
HA-r5E	HA	GTGGCTCTAATTCTGGGGCGGC GATTGGTTGGGGATCAGC	GCTGATCCCCAACCAATCGCCGC CCCAGAATTAGAGCCAC
HA-r18E	HA	GAGGGCGAAAATGAGGGGGCG GCGATTGGTTGGGGAGAAGC	GCTTCTCCCCAACCAATCGCCGC CCCCTCATTTTCGCCCTC
HA-r5A	HA	GTGGCTCTAATTCTGGGGCGGC GATTGGTTGGGGATCAGC	GCTGATCCCCAACCAATCGCCGC CCCAGAATTAGAGCCAC
HA-r18A	HA	CTGGCGCAAATGCTGGGGCGG CGATTGGTTGGGGAGCAGC	GCTGCTCCCCAACCAATCGCCGC CCCAGCATTTGCGCCAG

Table S7. List of primary and secondary antibodies used for IF

Name	Species	Epitope	Dilution	Reference
anti-TDP43	Mouse	AA 1-260 (N-ter)	IF (1:1000)	mAb ABnova H00023435-M01
anti-TDP43	Rabbit	(C-ter)	IF (1:1000)	pAb Proteintech 12892-1-AP
anti-HA	Mouse	HA tag (YPYDVPDYA)	IF (1:1000)	mAb Santa Cruz Biotechnology Sc-7392
anti-HA	Rabbit	HA tag (YPYDVPDYA)	IF (1:1000)	pAb Proteintech 51064-2-AP
anti-pTDP43 (S409/410)	Rabbit	Ser409/Ser410	IF (1:3000)	pAb Proteintech 22309-1-AP
anti-α-Tubulin	Mouse	AA 426-430 (C-ter)	IF (1:1000)	mAB Sigma-Aldrich T5168

Table S8.

List of secondary antibodies used for IF

Name	Species	Epitope	Dilution	Reference
Alexa Fluor 594	Mouse	IgG	IF (1 :1000)	Invitrogen A11005
Alexa Fluor 488	Rabbit	IgG	IF (1 :1000)	Invitrogen A11008
IR Dye 680 RD	Rabbit	IgG	IF (1 :1000)	LI-COR 926-68071

Table S9.

List of kinase inhibitor names and their target

Number	Name	Target
1	PD98059	MEK
2	U0126	MEK
3	SB 203580	p38 MAPK
4	H-7	PKA, PKG, MLCK
5	H-9	PKA, PKG, MLCK, PKC
6	Staurosporine	Pan-specific
7	AG 494	EGFRK, PDGFRK
8	AG 825	HER1-2
9	Lavendustin A	EGFRK
10	RG-1462	EGFRK
11	Tyrphostin 23	EGFRK
12	Tyrphostin 25	EGFRK
13	Tyrphostin 46	EGFRK
14	Tyrphostin 47	EGFRK
15	Tyrphostin 51	EGFRK
16	Tyrphostin 1	Negative control for tyrosine kinase
17	Tyrphostin AG 1288	Tyrosine kinases
18	Tyrphostin AG 1478	EGFRK
19	Tyrphostin AG 1295	Tyrosine kinases
20	Tyrphostin 9	PDGFRK

21	PKC 412	PKC
22	Piceatannol	Syk
23	PP1	Src family
24	AG-490	JAK-2
25	AG-126	IRAK
26	AG-879	NGFRK
27	LY 294002	PI 3-K
28	Wortmannin	PI 3-K
29	GF 109203X	PKC
30	Hypericin	PKC
31	Ro 31-8220 mesylate	PKC
32	D-Erythro-Sphingosine	PKC
33	H-89	PKA
34	HA-1004	PKA, PKG
35	HA-1077	PKA, PKG
36	2-Hydroxy-5-(2,5-dihydroxybenzyl amino) benzoic acid	EGFRK, CaMK II
37	KN-62	KN-62
38	KN-93	KN-93
39	ML-7	MLCK
40	ML-9	MLCK
41	2-Aminopurine	p58 PITSLRE beta1

42	N9-isopropyl olomoucine	CDK
43	Olomoucine	CDK
44	Iso-olomoucine	Negative control for olomoucine
45	Roscovitine	CDK
46	5-iodotubericidin	ERK2, CK1, CK2
47	LFM-A13	BTK
48	SB-202190	p38 MAPK
49	PP2	Src family
50	ZM-336372	cRAF
51	SU 4312	Flk1
52	AG 1296	PDGFRK
53	GW 5074	cRAK
54	Palmitoyl-D L-carnitine	PKC
55	Rottlerin	PKC delta
56	Genistein	Tyrosine kinases
57	Daidzein	Negative control for genistein
58	Erbstatin analog	EGFRK
59	Quercetin 2H2O	PI 3-K
60	SU1498	Flk1
61	ZM 449829	JAK-3

62	BAY 11-7082	IKK pathway
63	5,6-dichloro-1-β-D-ribofuranosylbenzimidazole	CK II
64	2,2′,3,3′,4,4′-Hexahydroxy-1,1′-biphenyl-6,6′dimethanol dimethyl ether	PKC alpha, PKC gamma
65	SP 600125	JNK
66	Indirubin	GSK-3beta, CDK5
67	Indirubin-3'-monooxime	GSK-3beta
68	Y-27632	ROCK
69	Kenpaullone	GSK-3beta
70	Terreic acid	Btk
71	Triciribin	Akt signaling
72	BML-257	Akt
73	SC-514	IKK2
74	BML-259	Cdk5/p25
75	Apigenin	CK-II
76	BML-259	Cdk5/p25
77	Rapamycin	mTOR
78	H-89	PKA
79	AG-370	PDGFRK
80	HNMPA	Tyrosine kinases
81	Y-27632 dihydrochloride	ROCK

82	Imatinib mesylate	BCR-ABL
83	Sorafenib tosylate	Tyrosine kinases
84	TC-S 7004	DYRK1A/B
85	JNJ-7706621	CDK1/2
86	C16 (PKRi)	PKR
87	CX-4945	CK2
88	SR-3029	CK1δ/ε
89	GSK-3 inhibitor XVI	GSK-3
90	IC261	CK1δ/ε
91	Binimetinib	MEK
92	Ibrutinib	BTK
93	PF-670462	CK1δ, p38, EGFR
94	BMS-536924	IGF-1R/IR
95	AC-480	HER1/2
96	Enzastaurin	PKC
97	Linifanib	VEGFR/PDGFR
98	Saracatinib	BTK, EGFR, Src

Table S10.

List of phosphatase inhibitor names and their target

Number	Name	Target
1	Cantharidic acid	PP2A
2	Cantharidin	PP1/2
3	Endothall	PP2A
4	L-p bromotetramisole oxalate	Alkaline phosphatase
5	RK-682	PTP1B
6	LEVAMISOL	Alkaline phosphatase
7	TETRAMISOLE	Alkaline phosphatase
8	CYPERMETHRIN	PP2B
9	Deltamethrin	Calcineurin (PP2B)
10	Fenvalerate	Calcineurin (PP2B)
11	Tyrphostin 8	Calcineurin (PP2B)
12	CinnGel	PTP1B
13	NSC-95397	Cdc25
14	BN-82002	Cdc25
15	Shikonin	PTP1B
16	NSC-663284	Cdc25
17	Cyclosporin A	PTP1B
18	Pentamidine	PRL

19	BVT-948	PTP
20	B4-Rhodanine	PRL3
21	Alexidine	PTPMT1
22	9,10-Phenanthrene -quinone	CD45
23	BML-260	DUSP22
24	Sanguinarine chloride	PP2C
25	BML-267	PTP1B
26	OBA	Tyrosine phosphatases
27	OBA ester	Tyrosine phosphatases (cell permeable)
28	Gossypol	5α-reductase 1 and 3α-hydroxysteroid dehydrogenase
29	Vorinostat	HDAC
30	Citicoline	Alkaline phosphatase
31	ZINC253496134	PTP1B
32	Tacrolimus	PTP1B
33	Alendronate	Tyrosine phosphatases
34	RWJ-60475	CD45
35	benzylphosphonic acid	PTP
36	RWJ-60475 (AM)3	CD45
